# How cryoEM has advanced our understanding of bacteriophages and bacteriocins targeting *Clostridioides difficile*

**DOI:** 10.1107/S2052252526005105

**Published:** 2026-06-23

**Authors:** Per A. Bullough, Jason S. Wilson, Hannah L. Berry, Robert P. Fagan

**Affiliations:** a Molecular Microbiology and Florey Institute of Infection, School of Biosciences University of Sheffield Firth Court, Western Bank SheffieldS10 2TN United Kingdom; Max Planck Institute of Molecular Physiology, Germany

**Keywords:** S-layers, cryoEM, phages, *Clostridioides difficile*, contractile injection systems, diffocins, receptor-binding proteins

## Abstract

We review the structural and functional characteristics of bacteriophages and bacteriocins (diffocins) that specifically target *Clostridioides difficile*, a significant healthcare concern due to its role in nosocomial infections. The advent of modern cryogenic electron microscopy (cryoEM) has revolutionized our understanding of these contractile injection systems, providing high-resolution insights into their mechanisms. We compare the structures of *C. difficile* phages and diffocins, highlighting their adaptations for penetrating the Gram-positive bacterial cell envelope – including the cell membrane, cell wall and protein­aceous surface layer. Diffocins, simpler in structure, utilize a combination of mechanical and enzymatic strategies, while some phages like ΦCD508 may rely on mechanical force alone. This review delves into the assembly and function of key components such as the contractile sheath, baseplate and receptor-binding proteins, offering a framework for engineering precision antimicrobials. We also present new experimental results, including refined cryoEM structures of the ΦCD508 pre- and post-contracted tail, a novel spontaneously contracted conformation and an X-ray crystal structure of a phage receptor-binding protein domain. This work underscores the potential of cryoEM in advancing our understanding of phage biology and its applications in developing targeted therapies against *C. difficile*.

## Abstract 2

Two recent cryogenic electron microscopy (cryoEM) structures provide a unique opportunity to compare a bacteriophage and a bacteriocin targeted at the same host. CryoEM reveals the most detailed view of contractile injection systems interacting with *Clostridioides difficile*.

## 1. Introduction

### 1.1. Structural biology of bacteriophages and contractile injection systems

Bacteriophages, henceforth termed ‘phages’, played a critical role in the early development of biological electron microscopy (EM), with Helmut Ruska, brother of the inventor of the electron microscope, Ernst Ruska, publishing the earliest EM images demonstrating the particulate nature of viruses, which in the case of bacteriophages showed distinctive heads and tails (Ruska, 1941 ▸). Luria and Anderson pioneered the use of EM techniques combined with shadowing and freeze-drying to better visualize phages, including the T-even series (T2, T4, T6). Their work was instrumental in defining the T-even morphology: a polyhedral head and a distinct tail structure (Luria *et al.*, 1943 ▸; Luria & Anderson, 1942 ▸). The introduction of the negative staining technique by Brenner, Horne and others greatly improved the resolution of EM images, allowing for detailed visualization of the T-even head and contractile-tail sheath (Brenner *et al.*, 1959 ▸). In the 1960s and 1970s the work of Klug, Caspar, DeRosier, Amos, Crowther and others established the three-dimensional organization, structural changes and assembly mechanisms of the helical phage tail, polyhedral viral capsids and baseplate using EM and image reconstruction techniques (De Rosier & Klug, 1968 ▸; Amos & Klug, 1975 ▸; Crowther *et al.*, 1977 ▸, 1970 ▸; DeRosier & Moore, 1970 ▸). Through the principle of quasi-equivalence, Caspar and Klug also found the key to building a large closed symmetric protein shell (capsid) from many copies of a single (or very few) type(s) of identical protein subunit(s), using a minimum amount of genetic information (Caspar & Klug, 1962 ▸).

Until recently, atomic resolution has only been achievable through X-ray crystallography of individual protein components of phages (Wikoff *et al.*, 2000 ▸; Simpson *et al.*, 2000 ▸), in some cases combined with low-resolution EM (Kostyuchenko *et al.*, 2005 ▸; Aksyuk *et al.*, 2009 ▸). The complexity and sheer size of an intact phage particle, and the difficulty in crystallizing it, limited this technique’s ability to solve the full whole-virion structure in atomic detail. Now, though, with the coming of the ‘cryoEM resolution revolution’ we are seeing a mini boom in high-resolution phage structures, including many in the last year (Wilson *et al.*, 2025 ▸; Hodgkinson-Bean *et al.*, 2025 ▸; Zhou *et al.*, 2025 ▸; Zhang *et al.*, 2025 ▸; Chen *et al.*, 2025 ▸; Hou *et al.*, 2025 ▸).

Many phages (and most of those with published structures) have contractile tails that help drive the phage genomic DNA into the host. These myophages are members of a larger family of contractile injection systems (CISs) that have a variety of biological roles and for which cryogenic electron microscopy (cryoEM) structures are also emerging (Cai *et al.*, 2024 ▸; Ge *et al.*, 2015 ▸; Kudryashev *et al.*, 2015 ▸; Jiang *et al.*, 2019 ▸; Desfosses *et al.*, 2019 ▸; Marín-Arraiza *et al.*, 2025 ▸). CISs that target Gram-positive bacteria must overcome the substantial barrier posed by their thick peptidoglycan cell wall, which typically ranges from 30 to 100 nm in thickness – markedly greater than the thin (∼2–5 nm) peptidoglycan layer of Gram-negative bacteria; this substantial barrier is further complicated by the presence of a shell of paracrystalline protein (the surface or S-layer) coating the surface of many bacteria. Whether this requires specialized penetration strategies is unclear, although we previously noted that CISs targeting Gram-positive bacteria enveloped by an S-layer appear to adapt their tail length to different hosts, while always appearing to extend the same length of inner tube (Wilson *et al.*, 2025 ▸). Systems directed against Gram-positive hosts also frequently incorporate a peptidoglycan hydrolase module to presumably aid penetration of the cell wall (Latka *et al.*, 2017 ▸).

### 1.2. *Clostridioides difficile* as a phage target

In this review, we will use the example of *Clostridioides difficile* (formerly *Clostridium difficile*) phages and bacteriocins to illustrate how the cryoEM resolution revolution is leading to a deeper understanding of the structure and host infection mechanism of CISs directed against a Gram-positive host. A compelling feature of *C. difficile* is that it is also enveloped by an S-layer (Fagan & Fairweather, 2014 ▸; Kirk, Banerji & Fagan, 2017 ▸; Lanzoni-Mangutchi *et al.*, 2022 ▸) (Fig. 7); S-layers present a formidable barrier to penetration by contractile-tail phages but, despite being nearly ubiquitous among bacteria and archaea, very few examples of S-layer penetrating CISs have been solved (Wilson *et al.*, 2025 ▸; Zhang *et al.*, 2025 ▸). CISs directed against *C. difficile* include both bacteriophages (Heuler *et al.*, 2021 ▸) and bacteriocins, known as ‘diffocins’; diffocins are bacteriocins that are produced by *C. difficile* strains to specifically kill other *C. difficile* strains. With the recent publication of both *C. difficile* phage and diffocin structures (Cai *et al.*, 2024 ▸; Wilson *et al.*, 2025 ▸) we now have the opportunity to directly compare two *C. difficile* infecting CISs, and relate similarities and differences to their adapted functions.

**Figure 1:**
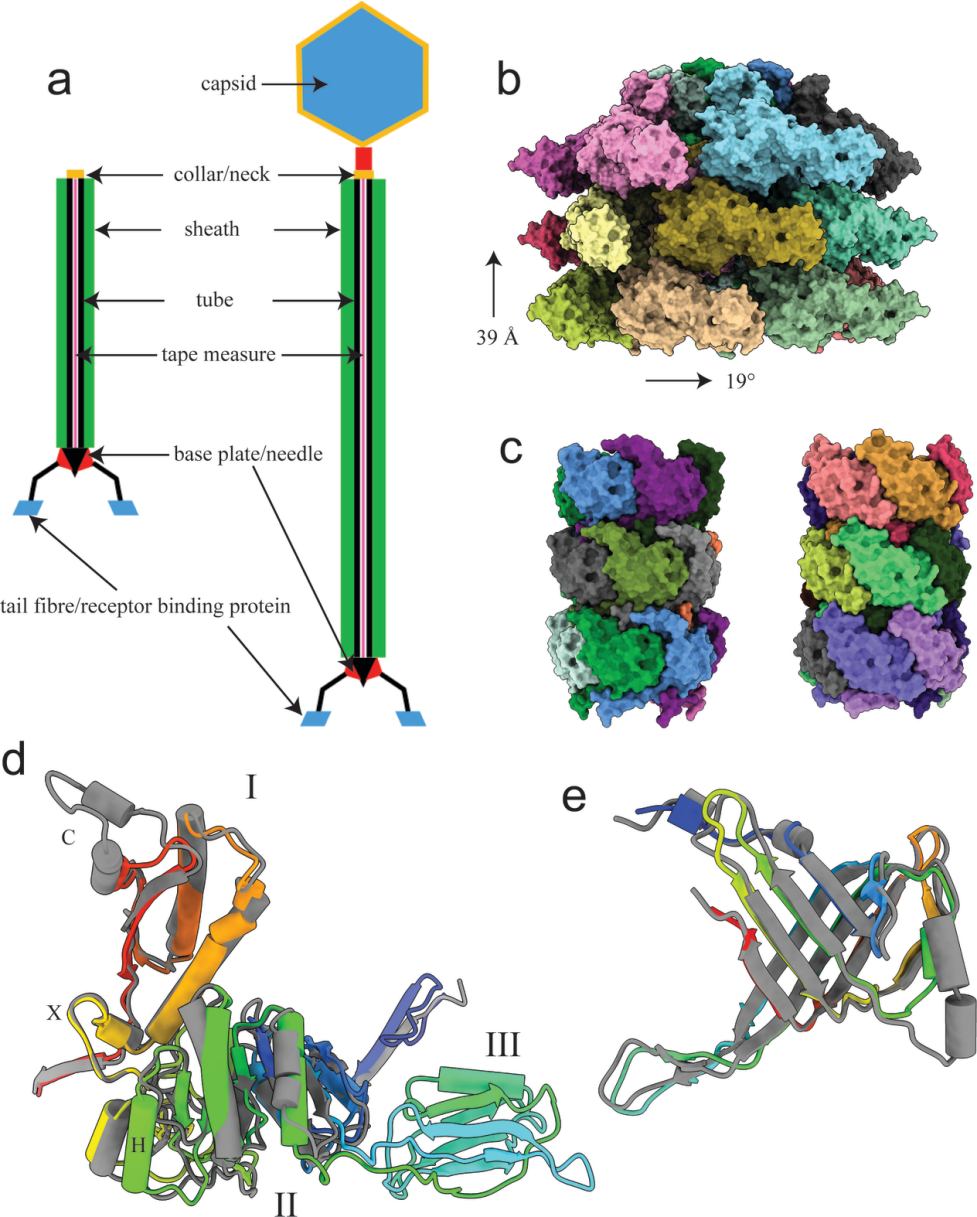
(*a*) Schematic figures of sections along the long axis of a diffocin (left) and contractile bacteriophage such as ΦCD508 (right). (*b*) Surface-rendered model showing three layers from the extended tail of ΦCD508 determined by Wilson *et al.* (2025 ▸). Sheath subunits are differentially coloured. (*c*) Three layers of the ΦCD508 tail tube (left), which has a similar architecture to that of an engineered diffocin, Av-CD291.2 (right). Individual subunits are differentially coloured. (*d*) Superposition of the main trunk sheath protein structures determined from pre-contracted forms of an engineered diffocin, Av-CD291.2 (grey, PDB ID 8v3x; Cai *et al.*, 2024 ▸), and phage ΦCD508 (rainbow coloured, N-terminus blue, C-terminus red). The ΦCD508 structure is a refined version of that originally described by Wilson *et al.* (2025 ▸), with additional residues at the N-terminus modelled in. Domains are labelled with Roman numerals; note that domain III in the phage sheath appears very disordered in the helical reconstruction, so the precise chain fold is poorly determined in this region. (*e*) Superposition of the tail tube subunit structures from pre-contracted forms of an engineered diffocin, Av-CD291.2 (grey, PDB ID 8v3x; Cai *et al.*, 2024 ▸), and phage ΦCD508 (rainbow, N-terminus blue, C-terminus red). The ΦCD508 structure is a refined version of that originally described by Wilson *et al.* (2025 ▸), with better fit of residues at the N- and C-termini. Figures made within *ChimeraX* (Meng *et al.*, 2023 ▸).

*C. difficile* is a significant healthcare concern, being the leading cause of nosocomial infection (Smits *et al.*, 2016 ▸; Buddle & Fagan, 2023 ▸). Disease is manifested as antibiotic-associated diarrhoea and, in severe cases, pseudomembranous colitis. Broad-spectrum antibiotics disrupt the gut microbial community, favouring the growth of *C. difficile*; thus, there is an urgent need for high-precision antimicrobials that selectively target this pathogen. Two candidates of interest have been phages (Nale *et al.*, 2016 ▸) and diffocins (Gebhart *et al.*, 2012 ▸; Kirk, Gebhart *et al.*, 2017 ▸). Phages employ specific mechanisms for host-cell recognition and either subsequent integration of their genome (lysogeny) or destruction of the host cell upon release of progeny virions (lysis). All *C. difficile* phages characterized to date are lysogenic. Diffocins, which are classified as phage tail-like bacteriocins (PTLBs), structurally and mechanistically mimic the contractile tails of certain phages (Schwemmlein *et al.*, 2018 ▸). However, unlike contractile-tail phages (myophages), diffocins lack a capsid and a genome [Fig. 1 ▸(*a*)].

This review is primarily concerned with a comparative analysis of published work on CISs targeting *C. difficile*. However, to augment this analysis we include some previously unpublished relevant methods and resultant data on tail contraction and receptor binding.

## 2. Materials and methods

### 2.1. Image processing of ΦCD508 tails

We reanalysed electron micrographs, previously analysed in Wilson *et al.* (Wilson *et al.*, 2025 ▸) (Fig. S1 of the supporting information). Briefly, movies were collected at 300 kV on a FEI Titan Krios with a Gatan K3 camera. Images were motion corrected in *MotionCor2* (Zheng *et al.*, 2017 ▸) and the contrast transfer function (CTF) estimated with *CTFFIND4* (Rohou & Grigorieff, 2015 ▸). Micrographs with a fitted CTF limit of 7 Å or poorer were discarded.

Template-free tracing in *FILAMENT TRACER* was used to select tail particles, with filament diameters set to 100–250 Å. Although the expected diameter of extended tails is ∼230 Å, these parameters allowed us to pick up the majority of unobstructed tails in each micrograph [Fig. 6(*a*), Fig. S1]. We used a separation distance of 39 Å, slightly greater than the spacing between stacked hexameric rings. An extraction box of 324 pixels (1.07 Å per pixel) covered a sufficient number of helical turns whilst minimizing the degradative effects of filament curvature when averaging particles. NCC (normalized cross correlation) and power scores were set to filter out ice contamination *etc*.

Two-dimensional class averaging was used to select ∼2.8 million particles, which were then subject to *ab initio* refinement with four starting classes and C6 symmetry imposition. This converged on four final volumes, two of which were uninterpretable, one representing the fully extended form of the tail (∼1.6 million particles) and one representing a previously unobserved state (∼0.8 million particles), but similar to the urea-induced fully contracted form (Wilson *et al.*, 2025 ▸), thus yielding two separate particle sets for further analysis [Fig. 6(*a*), Fig. S1].

The two selected volumes were used as starting models for refinement of the two respective particle sets in *HELIX REFINE*, with C6 symmetry but no helical parameters imposed. *SYMMETRY SEARCH* was performed on each reconstruction. We selected helical parameters that gave the minimum helical rise out of all valid transformations, thus ensuring maximum exploitation of the symmetry during averaging. Further cycles of *HELIX REFINE* enforced helical symmetry parameters and included local CTF refinement and the non-uniform refinement options.

The two reconstructions were used to generate templates for a second round of filament tracing (Fig. S1) to select ∼2.6 million particles, which were then subjected to 2D class averaging and *ab initio* refinement with three starting classes and C6 symmetry imposition. This converged to two volumes representing the extended tail and one volume representing the contracted tail. Particle sets from the two extended volumes were merged together and subjected to helical refinement (∼1.6 million particles, C6 symmetry, 38.9 Å rise and +19.2° twist). This yielded a ‘consensus’ reconstruction of the sheath and tube in the extended conformation. The third *ab initio* volume was used to produce a refined volume of the contracted sheath (∼770 thousand particles, C6 symmetry, 32.5 Å rise and +25.5° twist). As the inner tube did not follow the same helix, the density was smeared out; however, by enforcing the ‘extended’ helical parameters in refinement, we were able to separately recover the tube density (Fig. S1).

### 2.2. Model building and refinement

Atomic models were built and refined into the respective final volumes using a combination of *Coot* (Emsley & Cowtan, 2004 ▸), *Isolde* (Croll, 2018 ▸) and *Phenix* (Adams *et al.*, 2010 ▸) essentially as previously described (Wilson *et al.*, 2025 ▸).

### 2.3. 3D variability analysis of extended tails

Using the particle alignments from the ‘consensus’ sixfold symmetric reconstruction of the extended tail, we solved for three eigenvectors, followed by the generation of nine clusters, using 3D variability analysis (3DVA) (Punjani & Fleet, 2021 ▸) (Fig. S1). The resulting maps indicated large conformational variability in the sheath subunits – one such map (cluster 2) is shown in Fig. 5(*b*). However, several α-helices and the N-terminal region from residue 2 onwards were now much better resolved within individual cluster maps, allowing unambiguous fitting. Focusing on the central density ring, we remodelled two independent sheath subunits, fitting individual residues where possible. The density of domain III was still poor, so we performed a rigid-body fit of this domain using the *AlphaFold3* predicted structure [Fig. 5(*c*)]. This improved model corresponded approximately to a 3-start helix (C3 symmetry) with two independent subunits in the asymmetric unit, a helical rise of ∼39 Å and a left-handed twist of ∼41° (see Section 4.4).

### 2.4. Protein expression and purification of PtsM

Firstly, pET28a containing a C-terminally tagged full-length *ptsM* gene was used to overexpress PtsM protein. Protein was overexpressed in BL21 in 2xYT media. Cells were grown to ∼0.6 OD before inducing with 1 m*M* IPTG, and incubating overnight at 20°C. Cell pellets were harvested at 4500*g* for 20 min at 4°C.

Selenomethionine (SeMet)-derivatized protein was grown as above before induction, but once ∼0.6 OD was reached, cells were harvested and washed, and then resuspended in a minimal media consisting of 10.5 g l^−1^ K_2_HPO_4_; 1.0 g l^−1^ (NH4)_2_SO_4_; 4.5 g l^−1^ KH_2_PO_4_; 0.5 g l^−1^ trisodium citrate·2H_2_O; 5.0 g l^−1^ glycerol; 0.5 g l^−1^ each of adenine, guanosine, thymine and uracil; 1.0 g l^−1^ MgSO_4_·7H_2_O; 4.0 mg l^−1^ thiamine; 100 mg l^−1^ each of l-lysine, l-phenylalanine and l-threonine; 50 mg l^−1^ each of l-isoleucine, l-leucine and l-valine; and 40 mg l^−1^ seleno-l-methionine.

Native and derivatized PtsM was purified by resuspending ∼3 g cell pellets in 30 ml HisTrap buffer A (50 m*M* Tris pH 8, 300 m*M* NaCl). Cell suspension was sonicated, centrifuged for 15 min at 43 000*g*, and then filtered before loading on a 5 ml Histrap, pre-equilibrated in buffer A. PtsM was eluted by a 0–500 m*M* imidazole gradient in buffer A. Peak fractions were pooled, concentrated in a 10 kDa MWCO Vivaspin, and then loaded onto a Superdex 200pg column pre-equilibrated with 50 m*M* Tris pH 8 and 500 m*M* NaCl. Peak fractions were concentrated and stored at 4°C prior to crystallization.

### 2.5. Crystallization and structure determination of PtsM

Purified native and SeMet-derivatized PtsM was concentrated to 5 mg ml^−1^, and dialysed against 25 m*M* Tris pH 8 and 75 m*M* NaCl. Native crystals were grown in 0.1 *M* MgCl_2_, 0.1 *M* Na HEPES pH 7.5, and 10%(*w*/*v*) PEG 4000. Derivitized crystals were grown in 0.1 *M* BICINE pH 9.0, 2%(*v*/*v*) 1,4-dioxane, and 10%(*w*/*v*) polyethylene glycol 20 000. Prior to cooling in liquid nitrogen, crystals were cryoprotected in mother liquor containing an additional 20%(*v*/*v*) ethylene glycol.

Data for native and SeMet-derivatized crystals were collected at Diamond Light Source on beamline I03. Native crystals diffracted to 1.42 Å, and diffraction intensities were integrated and scaled using *FastDP*, space group *C*_2_ (Winter & McAuley, 2011 ▸). The SAD peak dataset used to phase the data was collected from the derivatized crystal at 0.9794 Å wavelength. Images were integrated and scaled using *FastDP*, to a resolution of 1.92 Å, also in space group *C*_2_.

Heavy-atom sites and an initial map were calculated by the *FastEP* pipeline. The asymmetric unit contained two chains of PtsM, and the map was of sufficient quality for initial model building using *Buccaneer* (Cowtan, 2006 ▸), and this initial model was used to calculate phases for the higher-resolution native dataset by molecular replacement, with a TFZ of 105, indicating a strong solution. The Matthews coefficient suggested that the unit cell contained <1 full protein chain. Protein stored at 4°C during crystallization was sent for LC–MS (liquid chromatography–mass spectrometry) analysis, and was shown to have spontaneously protealysed to form two smaller products. We confirmed this happened during crystallization by SDS–PAGE and mass-spectrometry analysis of freshly produced protein, both of which confirmed full-length protein. The final model in the native dataset was completed by manual building using *Coot*, and to a final *R* and *R*_free_ of 0.18 and 0.20, respectively.

## 3. Diffocins: structure, assembly and mechanism

We first consider diffocins, as these are less complex in structure than phages. Diffocins are high-molecular-weight bactericidal agents produced by specific *C. difficile* strains and targeted against competitor strains; they presumably play a role in sibling niche competition, but this has not been experimentally confirmed (Gebhart *et al.*, 2012 ▸). Expression can follow cellular stress, such as through the induction of the SOS response. Diffocins are analogues of the R-type pyocins found in organisms such as *Pseudomonas aeruginosa* (Ge *et al.*, 2020 ▸), which have been proposed to have evolved from defective bacteriophages (Nakayama *et al.*, 2000 ▸).

Diffocins function as molecular machines, designed to initiate rapid cell death, acting as a rigid contractile protein complex that penetrates the cell envelope. The mature particle is an ∼145 nm long rod [Fig. 1 ▸(*a*)], structurally organized around three primary elements: a baseplate, receptor-binding tail fibres, and a contractile sheath-and-tube structure wrapped around a core tape-measure protein (Schwemmlein *et al.*, 2018 ▸; Cai *et al.*, 2024 ▸; Scholl, 2017 ▸). The process of killing begins when a specific receptor-binding protein (RBP) binds to the major S-layer protein SlpA (Fig. 7). This binding is thought to act as a trigger, inducing an irreversible conformational change, via changes in the baseplate, that leads to the contraction of the outer sheath (Kirk, Gebhart *et al.*, 2017 ▸; Cai *et al.*, 2024 ▸). This contraction forcefully drives the inner tube (composed of CD1364 subunits) through the cell envelope, penetrating the S-layer and the peptidoglycan cell wall. The cell membrane is ultimately breached, leading to the lethal dissipation of the electrochemical membrane potential.

### 3.1. The contractile sheath/tube assembly

Diffocin contraction relies on two principal components: the outer sheath and the inner tube [Fig. 1 ▸(*a*)]. The structure of this assembly has been determined in the group of Hong Zhou (Cai *et al.*, 2024 ▸). The sheath protein (CD1363) forms the exterior contractile layer. The sheath protein is highly conserved compared with its phage tail-like relatives. However, CD1363 contains only two domains, a reduction compared with related phage tail sheath proteins, which typically comprise up to four domains [Figs. 1 ▸(*b*) and 1 ▸(*d*)]. This structural minimization suggests that R-type PTLBs, as exemplified by diffocins, represent the minimal protein architecture necessary to form a complete and functional contractile sheath (Schwemmlein *et al.*, 2018 ▸); this raises the question of what the additional domains in phages are for (see below).

The inner tube protein (CD1364) forms a rigid rod-like inner core [Figs. 1 ▸(*a*), 1 ▸(*c*) and 1 ▸(*e*)]. This inner structure is driven across the host-cell envelope upon sheath contraction (Cai *et al.*, 2024 ▸). Both the tube and sheath complexes are formed of discs that assemble from six sheath or tube subunits via an axial sixfold rotational symmetry and are stacked atop each other after rotation. However, the sheath protein adopts two different conformations along the main tail length, in alternating layers (Cai *et al.*, 2024 ▸). An analogous phage tail, with a similar sheath and tube arrangement of sixfold discs is shown in Fig. 1 ▸(*b*).

The transition from the monomeric pre-assembly states of the tube and sheath [determined by X-ray crystallography (Schwemmlein *et al.*, 2018 ▸)] to the final functional tail complex [determined by cryoEM (Cai *et al.*, 2024 ▸)] necessitates significant conformational changes. For the sheath protein (CD1363), the rearrangement of the flexible N- and C-termini is critical, enabling the extensive inter-subunit interaction network required for stable assembly and concerted contraction.

### 3.2. The baseplate and collar

**Figure 2:**
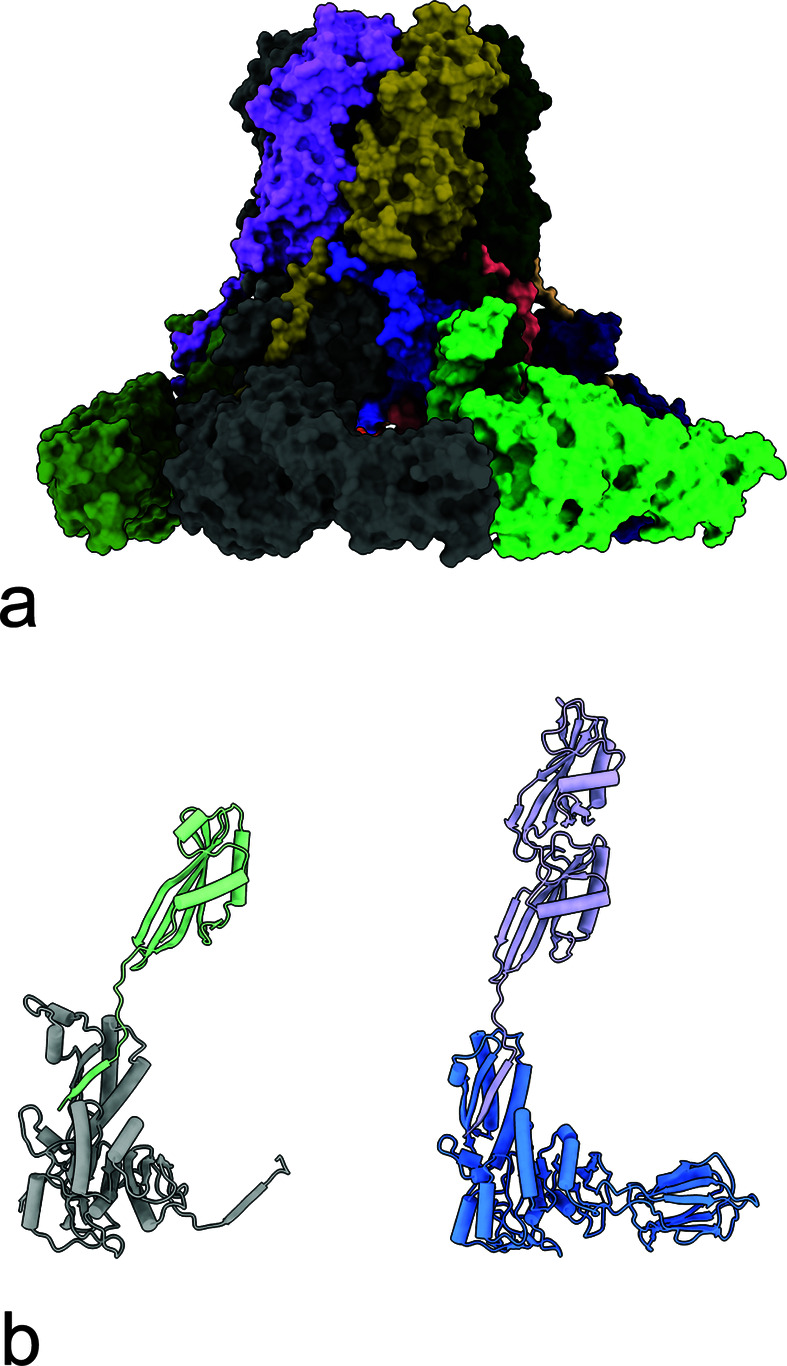
(*a*) Surface-rendered model of the tail terminator/sheath assembly in ΦCD508 with subunits differentially coloured. The arrangement in the diffocin collar is similar but both collar (equivalent to the ΦCD508 tail terminator) and sheath subunits have fewer domains – see (*b*). (*b*) Individual collar/sheath components of ΦCD508 (right) and Av-CD291.2 (left). The ΦCD508 tail terminator protein (mauve) has an additional domain compared with the collar protein of Av-CD291.2 (pale green); PDB IDs 9gb7 (Wilson *et al.*, 2025 ▸) and 8v3t (Cai *et al.*, 2024 ▸).

The collar and baseplate are key components for contraction. The collar [Fig. 1 ▸(*a*)] functions as a force transducer between sheath contraction and tube ejection. It is composed of a hexameric ring of collar protein CD1362 [Fig. 2 ▸(*b*)] that bridges the tube and sheath. The N-terminal domain of the collar subunit binds to the tube, while its C-terminal β-strand extends towards the C-terminal β-hairpin of the last sheath subunit, forming a three-β-strand handshake interaction. These interfaces are maintained during contraction, enabling the collar to effectively transmit the contraction force.

The baseplate [Figs. 1 ▸(*a*) and 3 ▸(*a*)] has an inner section that connects to the tube and an outer section that connects to the sheath. The inner section comprises three proteins: tube tail (CD1367; also known in the literature as ‘tail tube initiator’), hub-hydrolase (CD1368) and spike (CD1369). Six copies of the tube tail form a ring immediately below the first ring of the core tube. Three copies of the hub-hydrolase sit below the tube tail, enclosing the N-terminal α-helices of the spike trimer. The outer section consists of sheath initiator (CD1370) and triplex proteins (CD1371 and CD1372). Two conformers of CD1371, Tri2A and Tri2B, and one of CD1372, Tri1, form the triplex, and six copies of the triplex form the baseplate wedge, surrounding the inner baseplate. The sheath initiator acts as an intermediate between the triplex and the first layer of the sheath, and between the tube tail and the hub-hydrolase. The baseplate wedge features a hexagonal iris-like architecture formed by the heterotrimeric triplex complexes (Tri2A–Tri2B–Tri1).

The C-terminal region of Tri1, along with the tail fibre linked to it, is not resolved in the cryoEM maps, but it is implied that the tail fibres are connected to the baseplate through this triplex structure. The specific attachment of the tail fibres to receptors on the target bacterial cell surface is crucial for triggering the conformational changes in the baseplate that lead to the contraction of the sheath and the injection of the tube. The baseplate reorganization, triggered by RBP binding, must drive two concurrent mechanical processes: (1) initiating the collapse of the metastable sheath and (2) releasing the sequestered CD1368 hub-hydrolase.

### 3.3. The tape-measure protein

The tape-measure protein (TMP) (Cai *et al.*, 2024 ▸) determines the length of the diffocin. It is located in the lumen of the central tube and spans the entire length of the particle [Fig. 1 ▸(*a*)]. The TMP assembles as a coiled-coil trimer, with its C-terminal α-helices interacting with the N-terminal α-helices of the spike trimer, forming an intertwined helix bundle; the N-termini of the TMP interact with the collar.

In addition to determining the length of the diffocin, the TMP may have other potential functions. Structure prediction tools suggest that the TMP contains a globular domain and a transmembrane (TM) region (Cai *et al.*, 2024 ▸). The globular domain is predicted to be composed of a series of short α-helices, while the TM region is predicted to form multiple TM helices. These features suggest that upon ejection from the tube, the TMP may refold to form a sizable pore on the membrane of the target cell, dissipating the electrochemical gradient and contributing to the bactericidal action of the diffocin.

## 4. Structure of a *C. difficile* phage, ΦCD508, and comparison with the diffocin structure

All currently isolated *C. difficile* phages belong to the *Caudoviricetes*-tailed phages, with dsDNA genomes (Nale *et al.*, 2022 ▸, 2012 ▸; Fortier & Moineau, 2007 ▸). These were traditionally classified into two major families: myoviridae, characterized by a long rigid tail equipped with a contractile sheath, and siphoviridae, which possess a long, often flexible, but non-contractile tail. This review will focus on a *C. difficile* phage with a contractile tail, as structural information on non-contractile *C. difficile* phages is limited (Dowah *et al.*, 2021 ▸).

Phages are fundamentally distinct from diffocins because they contain a capsid, which is the protein shell housing the dsDNA genome [Fig. 1 ▸(*a*)]. The tail is the complex apparatus responsible for host infection and genome delivery, including the portal, neck and tail proteins (Wilson *et al.*, 2025 ▸), and in myoviridae is structurally analogous to the contractile bacteriocins [Fig. 1 ▸(*a*)]. All known *C. difficile* phages are temperate, meaning their genomes encode integrase genes, allowing them to adopt a lysogenic life cycle in which the genome (prophage) is integrated into the host chromosome rather than immediately entering a replicative cycle that leads to the lysis of the infected cell (Umansky & Fortier, 2023 ▸).

### 4.1. Overall structure

We recently published the first structure for an intact *C. difficile* phage, ΦCD508 (Wilson *et al.*, 2025 ▸) [Fig. 1 ▸(*a*)]; this gives us a rare opportunity to directly compare the structure and mechanism of action of a bacteriocin and a bacteriophage directed at the same bacterial host. Moreover, we have also recently determined the structure of the common SlpA receptor for diffocins and ΦCD508 (Lanzoni-Mangutchi *et al.*, 2022 ▸), opening up future opportunities for detailed mechanistic interrogation of receptor binding and infection initiation (see below).

**Figure 3:**
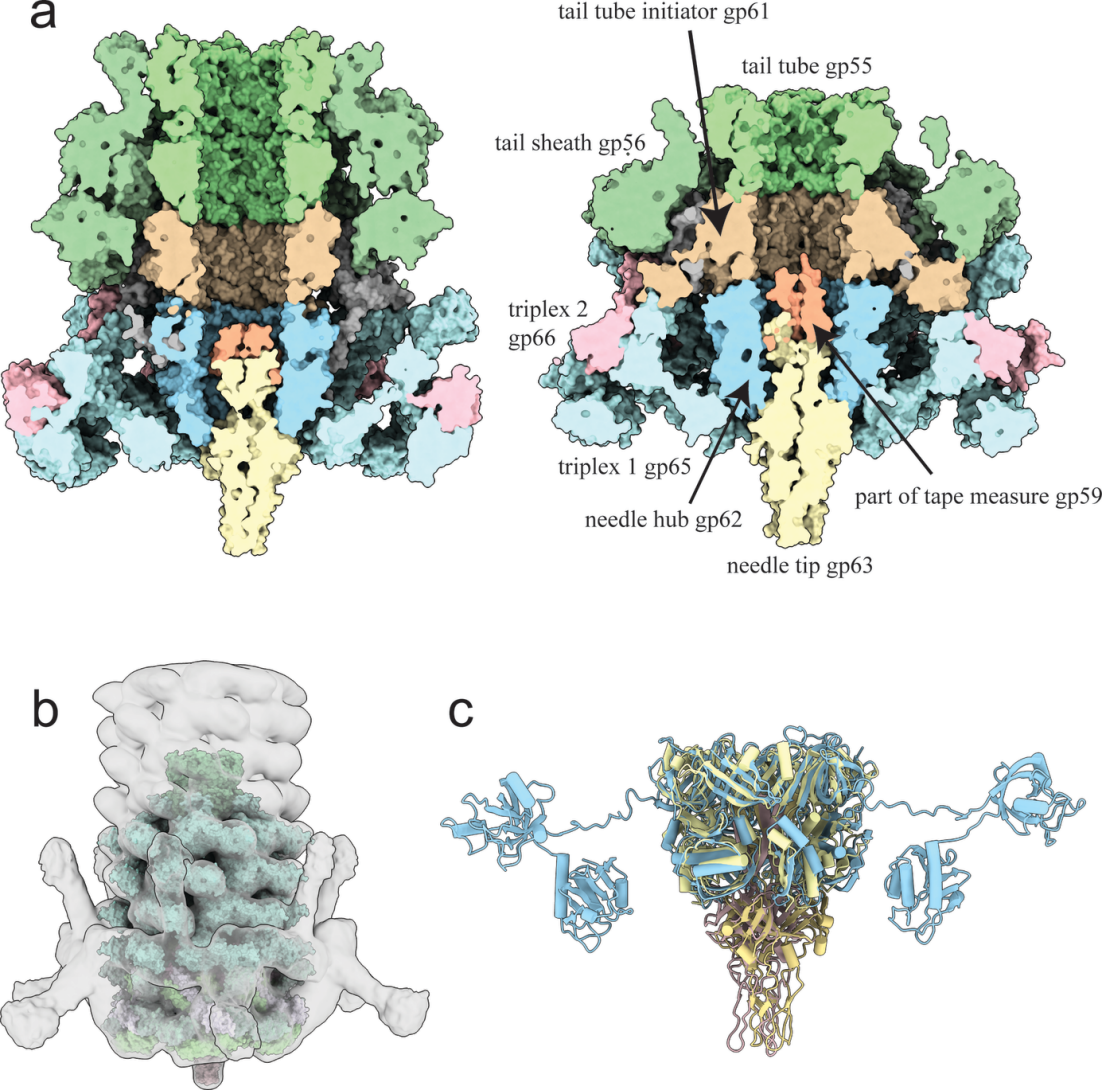
(*a*) Sectioned surface-rendered models of baseplate and needle of ΦCD508 (right) and Av-CD291.2 (left). Analogous or homologous subunits are coloured the same in the two structures. (*b*) Low-pass filtered cryoEM reconstruction of ΦCD508 baseplate and terminal region of the tail. An atomic model of the baseplate hub, wedge and tail components is shown, highlighting the unfitted density corresponding to the attached tail fibres. (*c*) Overlay of ΦCD508 and Av-CD291.2 hub and needle indicating structural homology of needle proteins and core domains of the hub protein. The additional hub-hydrolase domains of Av-CD291.2 (pale blue) are seen radiating out from the central axis.

**Figure 4:**
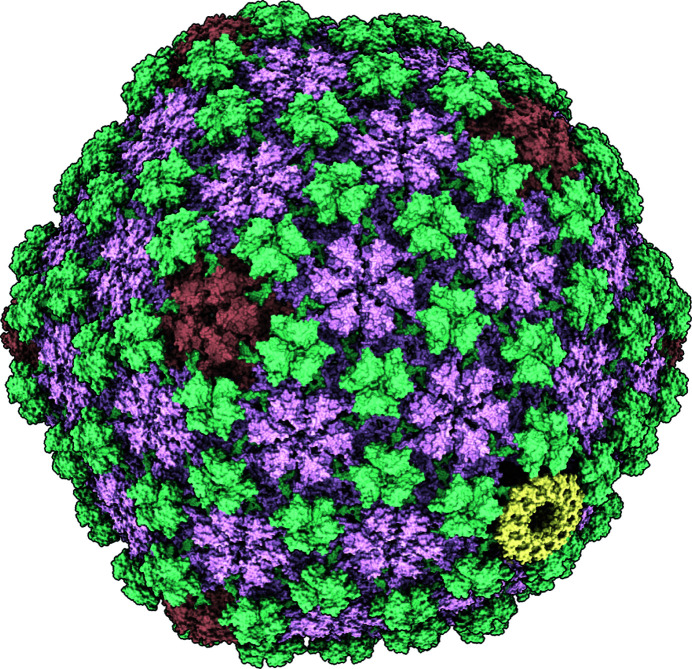
Surface-rendered model of ΦCD508 capsid. Major capsid protein gp49 subunits are arranged around icosahedral fivefold axes (brown) and around quasi-sixfold axes (plum). Minor capsid subunits, gp48, are arranged around threefold axes (green), One fivefold axis is occupied by a 12-fold symmetric portal (yellow).

The extended phage structure of ΦCD508 [Fig. 1 ▸(*a*)] consists of several distinct regions (Wilson *et al.*, 2025 ▸):

(1) Head: the head is a near-complete *T* = 7 (Caspar & Klug, 1962 ▸) icosahedral capsid with a diameter of 650 Å, housing the genomic DNA. It is composed of two capsomer proteins, the major capsid protein gp49 and the capsid decoration protein gp48. The capsid has a unique opening at the base, formed by 12 copies of the portal protein gp45, through which DNA is ejected (Fig. 4 ▸).

(2) Neck: the neck connects the capsid to the tail and is made up of three protein complexes. It includes a dodecameric ring (gp50) and two hexameric rings (gp51 and gp53). The neck features a narrow constriction formed by the neck valve protein gp51 (Fig. 2 ▸).

(3) Tail: the tail is unusually long at 225 nm [Fig. 1 ▸(*a*)] and consists of a stack of 57 nested hexameric rings. The inner ring is formed by the tail tube protein gp56, and the outer ring by the tail sheath protein gp55. The tail in its extended form was initially described as a six-stranded helix (C6 symmetry) with a twist of +19° and a rise of 39 Å (Wilson *et al.*, 2025 ▸) [Figs. 1 ▸(*b*) and 1 ▸(*c*)].

(4) Baseplate and needle: the baseplate is minimal compared with other phages and consists of six unique proteins organized into a baseplate hub and a wedge [Fig. 3 ▸(*a*)]. The needle assembly, formed by proteins gp62 and gp63, provides a pointed end terminating the tail. The needle is stabilized by a coordinated metal ion at its tip, putatively a ferrous ion [Fig. 3 ▸(*c*)].

### 4.2. Neck and collar compared in diffocin and ΦCD508

Diffocins do not have a DNA-containing capsid, and therefore no neck structure is required. However, supporting the hypothesis of an evolutionary relationship between phages and other CISs, the diffocin structure has a collar protein (CD1362), with structural homology to the ΦCD508 tail terminator, gp53 of the neck complex [Figs. 1 ▸(*a*) and 2 ▸(*b*)].

In both the diffocin and the phage, the collar/tail terminator serves as a structural component that links the sheath to the tube, playing a critical role in transmitting the contraction force [Fig. 1 ▸(*a*)]. In both cases, the ring is hexameric but this is effectively double-layered in the phage, with an extra domain of similar fold [Fig. 2 ▸(*b*)]. Also, in both cases, the β-sheet handshake interaction [Fig. 2 ▸(*b*)] between the collar and the sheath is a feature that ensures the mechanical connection to the sheath during contraction. This interaction allows the neck-proximal sheath protein to rotate and accommodate the contraction-induced movements of the other sheath proteins in the tail below, pivoting around a specific glycine residue (Gly260) in the gp53 C-terminal linker. No obvious equivalent candidate for a pivot is found in the diffocin structure, with the equivalent position being taken by a valine.

### 4.3. Baseplates compared in diffocin and ΦCD508

The baseplate of ΦCD508 [Fig. 1 ▸(*a*)] is constructed from six unique proteins that are organized into two main components: the baseplate hub and the wedge [Fig. 3 ▸(*a*)]. The baseplate hub connects to the tail, while the wedge forms a collar around the needle. The baseplate is also expected to include the host attachment proteins, such as the gp67 tail fibre (baseplate attachment) protein and RBP gp68, which are involved in binding to the host S-layer [Fig. 1 ▸(*a*)]. These proteins are not fully resolved in the EM maps, but their presence is suggested by protrusions observed through low-pass filtering [Fig. 3 ▸(*b*)]. The baseplates of both diffocins and phages serve as the initiation point for contraction, triggered by interactions with the target cell surface. In diffocins and ΦCD508, the baseplate includes a specialized central spike or needle, containing a stabilizing metal ion, but unlike diffocin, ΦCD508 does not have a hub-hydrolase protein [Fig. 3 ▸(*c*)].

For successful infection, the mechanical signal – stemming from irreversible RBP binding – must be transmitted from the periphery of the baseplate to the tail, ultimately triggering sheath contraction. The recent cryoEM study elucidating the firing mechanism of the *Algoriphagus machipongonensis* CIS (AlgoCIS) provides the first definitive evidence of a highly coordinated stepwise mechanical cascade governing CIS activation (Xu *et al.*, 2026 ▸). For *C. difficile*-associated CISs, we are so far missing this level of dynamic detail. However, the essential modules responsible for structural stability and signal transmission – the baseplate wedges – appear conserved in design. The modular organization maintains the CIS blueprint necessary for nucleating the tail tube and initiating sheath polymerization. This high degree of structural streamlining is in stark contrast to the *Escherichia coli* phage T4 baseplate, which has 15 different proteins and over 100 subunits (Taylor *et al.*, 2016 ▸; Crowther *et al.*, 1977 ▸). Thus, both ΦCD508 and diffocin achieve a complete functional contractile system with far fewer components.

The most striking difference in the baseplate assembly is in the protein connecting the tube to the needle tip; in the diffocin, this is identified as a hub-hydrolase, whereas in ΦCD508 we named it as a needle hub protein [Fig. 1 ▸(*a*)] (Cai *et al.*, 2024 ▸; Wilson *et al.*, 2025 ▸). In both particles, the needle spike/tip and the hub/hydrolase adopt very similar folds [Fig. 3 ▸(*c*)], with a putative metal ion stabilizing the needle tip in both cases. However, in the diffocin the hydrolase protein incorporates additional C-terminal enzymatic domains that are not present in ΦCD508. The presence of a bifunctional hydrolase domain in the diffocin’s hub-hydrolase protein (CD1368) is presumably an adaptation for degrading the thick peptidoglycan layer of the Gram-positive *C. difficile*. CD1368 has two distinct predicted enzymatic domains: a lytic transglycosylase and an endopeptidase. Thus, the protein is capable of cutting both the glycosidic linkages of glycan strands and the peptide bonds of oligopeptide chains within the peptidoglycan mesh. These combined activities would effectively degrade the thick cell wall, and presumably minimize the mechanical force required for envelope penetration. In striking contrast, the ΦCD508 system appears to rely entirely on mechanical energy for cell-envelope penetration – there are no obvious auxiliary domains with structural homology to peptidoglycan hydrolases in any of the ΦCD508 baseplate or needle proteins.

### 4.4. Sheath and tube structures compared; revisiting the image analysis

Tailed phages and bacteriocins are endowed with multiple symmetry elements from the typically threefold symmetric tail tip, through the usually sixfold helical symmetry of the main tail, and in the case of phages, to the often icosahedral or quasi-icosahedral symmetry of the DNA-containing capsid. These symmetries have been usefully exploited in single-particle analysis of phage images, but mismatches in symmetry between different components (fivefold, 12-fold, sixfold and threefold) mean that phage images cannot be reconstructed without judicious use of masking; thus, workflows are relatively complex (Cai *et al.*, 2024 ▸; Wilson *et al.*, 2025 ▸). An added challenge with such large multicomponent complexes is to identify and trace the densities for individual polypeptides; the application of machine learning in predicting three-dimensional folds from protein sequences has greatly simplified this task (Abramson *et al.*, 2024 ▸; Baek *et al.*, 2021 ▸). But this has also highlighted that AI structure predictions are far from infallible, with some phage subunits (*e.g.* baseplate triplex proteins and sheath handshake domains) [Figs. 6(*c*) and 6(*e*)] adopting multiple different conformations, not predicted by *AlphaFold* (Abramson *et al.*, 2024 ▸), for example (Wilson *et al.*, 2025 ▸).

All CIS sheath assemblies studied so far share a similar core fold, exemplified by the diffocin sheath [Fig. 1 ▸(*d*)]. The contractile sheath is almost invariably composed of six interacting helical protein strands that drive the injection process [Figs. 1 ▸(*b*) and 5 ▸(*d*)]. The sheath’s geometric parameters (diameter, helical rise and twist) are generally quite similar [Fig. 1 ▸(*b*)], though system-specific adaptations exist, such as the two-layered sheath in antifeeding prophage (Desfosses *et al.*, 2019 ▸) and widely varying tail lengths (Wilson *et al.*, 2025 ▸).

For the purposes of this review, we decided to revisit some of our image processing approaches since the publication of Wilson *et al.* (2025 ▸) – see Section 2. This was partly inspired by the finding that the diffocin sheath subunit (CD1363) adopts slightly different conformations in alternating hexameric rings along the tail (Cai *et al.*, 2024 ▸); correctly determining helical parameters can be difficult (Egelman, 2024 ▸; Gambelli *et al.*, 2022 ▸). We wished to test whether, by analogy, such an arrangement is also adopted in ΦCD508.

In our previous analysis of the ΦCD508 extended tail (Wilson *et al.*, 2025 ▸) we picked a relatively small (∼120 000) number of helical segments using a combination of *crYOLO*, *EMAN*, *RELION* and finally *cryoSPARC* (Wagner *et al.*, 2019 ▸, 2020 ▸; Tang *et al.*, 2007 ▸; He & Scheres, 2017 ▸; Punjani *et al.*, 2017 ▸) to pick particles and construct a map to 2.7 Å resolution; C6 symmetry and helical parameters of +19.2° twist and a 38.9 Å rise were enforced. We have now revisited these data, exploiting the *filament tracer* option within *cryoSPARC*. The main steps are described in Section 2 and Fig. S1.

**Figure 5:**
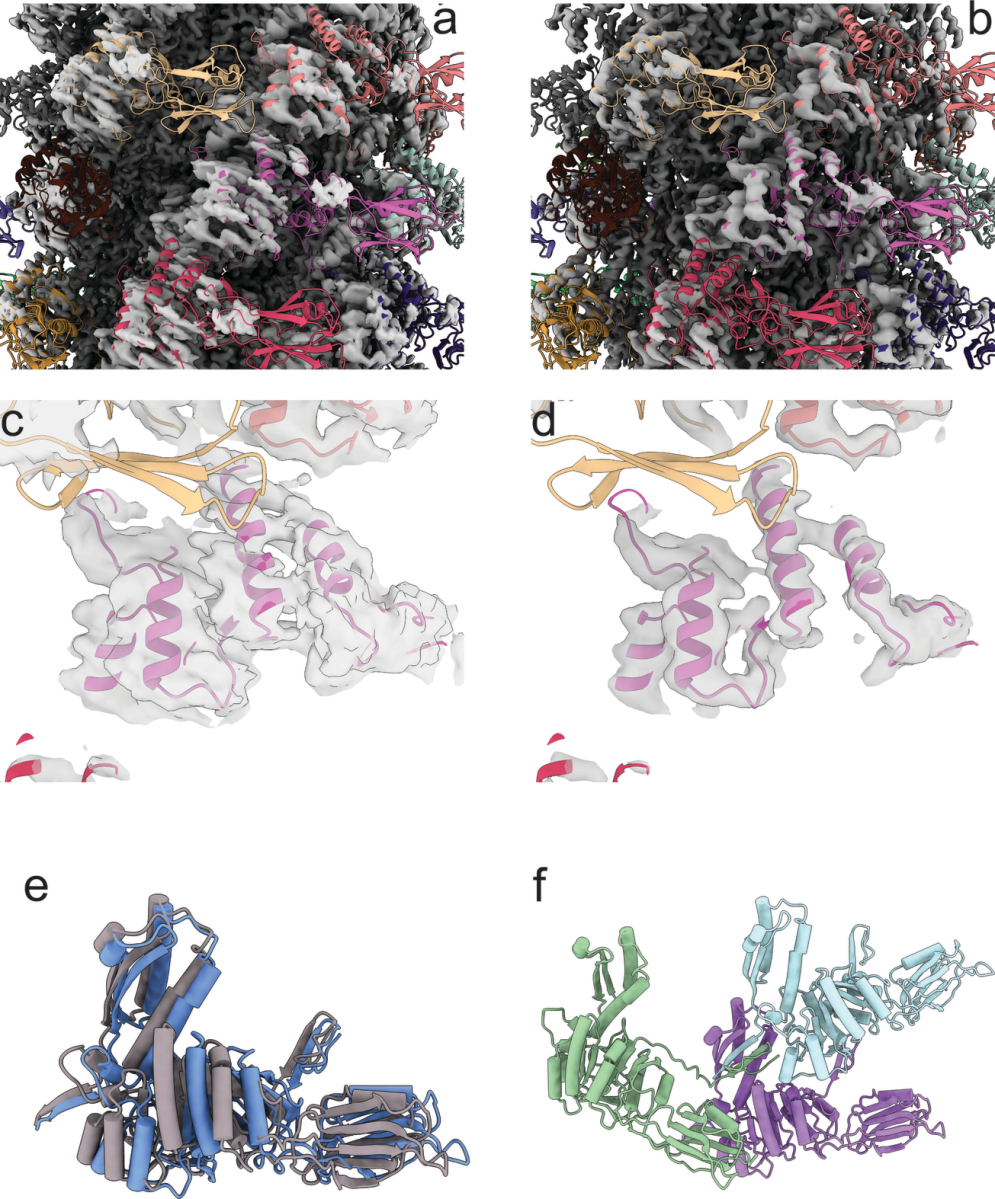
(*a*) Representative experimental cryoEM density from the improved resolution map (overall resolution 2.3 Å) of the ΦCD508 pre-contracted tail with helical symmetry imposed. The density threshold has been set to reveal domains I and II [see Fig. 1 ▸(*c*)] more clearly. Polypeptide backbones of individual sheath subunits are differentially coloured. (*b*) Representative density of reconstruction from one cluster of images selected after 3DVA of density in (*a*). Individual subunits have been fitted by rigid-body refinement followed by local refinement. (*c*) Closer view of segment of the central protomer from panel (*a*), with experimental density set to transparent. Density is smeared out roughly perpendicular to the long axes of the α-helices suggesting a superposition of multiple conformations of the helices. This is supported by the view in (*d*). (*d*) Density from a selected cluster of images subjected to 3DVA. Compared with (*c*), fits of the α-helices are much less ambiguous. (*e*) Comparison of two sheath subunits from the model in (*b*). Two adjacent subunits within the middle ring of the model have been superimposed after a relative rotation of 60° around the central tail axis. (*f*) Three adjacent subunits of the extended sheath. Green and purple are from one layer [see Fig. 1 ▸(*a*)] whilst the pale blue subunit is from an adjacent layer. All three subunits contribute strands to the central handshake.

By revisiting the image analysis, we were able to use ∼1.6 million particles in the extended tail reconstruction and ∼770 000 particles in the ‘contracted’ reconstruction. Thus, we extended the nominal resolution from 2.7 to 2.3 Å for the extended state and from 4.2 to 3.6 Å for the contracted states described previously by Wilson *et al.* (2025 ▸) (Figs. S1 and S2). We also obtained a separate structure for the tube of the contracted state at 2.3 Å resolution. A few points are also worth noting:

(i) By extracting many more particles than previously, and using multiple *ab initio* classes, we were able to separate out both fully extended particles and particles representing a ‘naturally’ contracted state (see below).

(ii) The ability to separate extended and contracted states was sensitive to the number of *ab initio* volumes; the reasons for this are unclear.

(iii) Whilst the resolution was clearly improved in well ordered regions of the structure, notably the tube and adjacent parts of the sheath, the outer domain III sheath and parts of domain II density remained very poor, as originally observed by Wilson *et al.* (2025 ▸) [Fig. 1 ▸(*d*), Fig. 5 ▸(*a*), Fig. S2]. The process of 3DVA proved a powerful approach to interrogate this problem (Punjani & Fleet, 2021 ▸) (see Section 2.3). One of the resultant cluster maps allowed us to build an improved model corresponding approximately to a 3-start helix (C3 symmetry) with two independent subunits in the asymmetric unit, a helical rise of ∼39 Å and a left-handed twist of ∼41° (Fig. 5 ▸). From this, we conclude that the helical tail is best described not as a regular 6-start helix but rather as adopting a continuum of different conformations, including one approximating a threefold symmetric tail with an asymmetric unit consisting of two independent sheath subunit orientations. Analysis of other phage tail structures may well benefit from this deeper interrogation – a simple global application of sixfold symmetry may not always be the best option.

(iv) In our original analysis (Wilson *et al.*, 2025 ▸) we were unable to resolve the tube structure in the contracted tail since this did not adopt the same helical symmetry as the sheath. We have now resolved this by enforcing the tube helical parameters (which remain unchanged after sheath contraction) during helical refinement. The fold is essentially identical in both states (Fig. S2).

(v) The improved resolution has revealed additional density in the vicinity of the side chain of Cys49 of the tube. This is independently verified in the ‘contracted’ tube density [Fig. S2(*f*)].

### 4.5. A novel conformational state for ΦCD508

**Figure 6:**
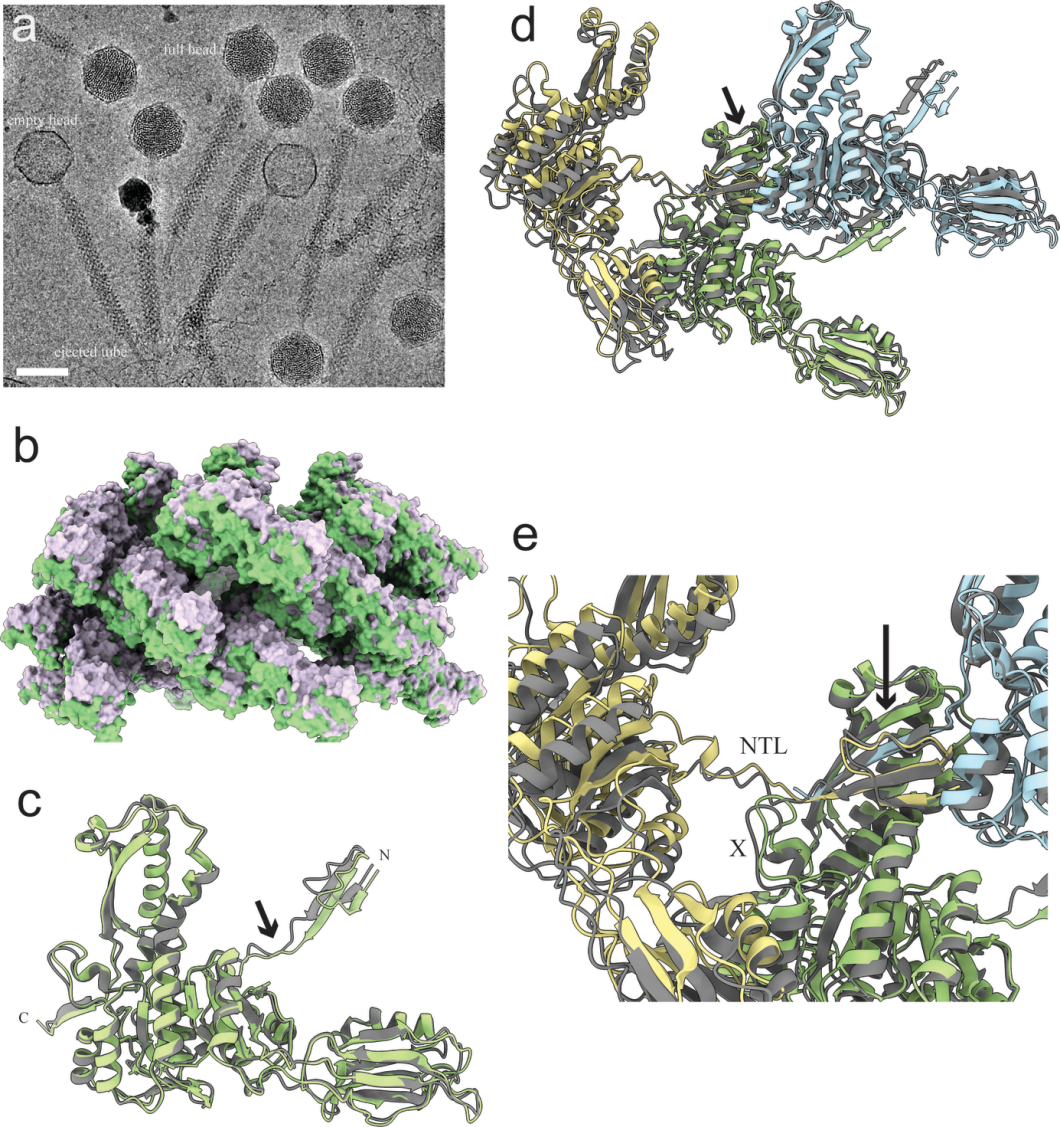
(*a*) Field of view of ice-embedded ΦCD508 particles showing how some tails have spontaneously contracted to eject part of the inner tube. In some contracted particles the capsid appears empty, whereas in others it appears to still contain DNA. Scale bar: ∼650 Å. (*b*) Comparison of surface models of urea-contracted ΦCD508 tail sheath (PDB ID 9gb6, mauve; Wilson *et al.*, 2025 ▸) and spontaneously contracted sheath (green), showing three layers. The structures were aligned on one subunit in the lower of the three layers. (*c*) Superposition of sheath subunit from reprocessed urea contracted tail (grey) and naturally contracted tail (pale green). The bulk of the three domains behave as a rigid body but flexibility in the N-terminus is indicated by the arrow. (*d*) Comparison of three neighbouring sheath subunits from urea-contracted ΦCD508 tail (grey) with the equivalent subunits from the naturally contracted tail (coloured). Polypeptide chains have been aligned on the β-handshake domain where three chains meet around the C-terminus of the pale blue subunit and the N-terminus of the straw subunit (arrow). (*e*) Detail from (*d*) showing the region around the aligned β-handshake domain (arrow). The N-terminal linker (NTL) is partially unfolded in the urea-contracted tail (grey). This accommodates a shift in the X-loop (X) and thus a rigid-body movement of the subunit as a whole.

Our reanalysis of the ‘pre-contraction’ particle sample described in the previous section detected a subset of particles in a contracted form (Fig. 6 ▸). Remarkably, this contracted form has a different overall conformation from the one calculated from particles incubated in a urea buffer (Wilson *et al.*, 2025 ▸) (Fig. 6 ▸; Table 1 ▸). Fig. 6 ▸ shows how the different conformations are accommodated by flexibility in the N-terminal linker, essentially pivoting around the tripartite β-sheet where three neighbouring subunits are tied together [Figs. 6 ▸(*c*)–6 ▸(*e*)]. Accompanying this rearrangement of the linker from one subunit is a shift in position of the X-loop forcing a repositioning of the entire neighbouring subunit. Whilst the resolution of the map in the linker region is too poor to reliably fit side chains, it seems likely that there is a reduction of the number of hydrogen-bonding interactions, perhaps brought on by partial denaturation induced by the urea. Thus, the collapse of the sheath in urea appears to be more extensive, raising the question of whether this is a real difference or simply reflects variability in the contracted state, as analysed for diffocin (Cai *et al.*, 2024 ▸). In either case, the improved resolutions for extended and contracted tails provide us with refined models for both extended tube/sheath and contracted sheath. These have all been used in the current analysis and comparisons with the diffocin structure.

### 4.6. Overall comparison of sheath subunit folds

Comparison of sheath subunits from ΦCD508 and diffocin shows the same basic fold of domains I and II [Fig. 1 ▸(*d*)]. However, there are some significant differences, for example, the additional domain III in ΦCD508, an inserted short helix in ΦCD508 [‘H’ in Fig. 1 ▸(*d*)] and an inserted α–α corner in diffocin [‘C’ in Fig. 1 ▸(*d*)]. The functional implications of these differences are best considered in the context of the whole sheath assembly [Figs. 1 ▸(*b*) and 6 ▸]. ΦCD508 shows an unusually small contraction, and we attributed this to the extended X-loop [Figs. 1 ▸(*d*), 6 ▸(*d*) and 6 ▸(*e*)] blocking full collapse of the sheath in the contracted state (Wilson *et al.*, 2025 ▸). While the contraction of diffocin is greater than that of ΦCD508, it is still relatively small (34%) compared with other CISs, which typically contract by about 50% of full length. Cai *et al.* (2024 ▸) attributed this reduced contraction to the presence of an α–α corner motif blocking complete collapse of the sheath [Fig. 1 ▸(*d*)]. However, we suggest that the equivalent of the ‘X-loop’ in the diffocin sheath protein may also be a contributing factor. There is no equivalent of the α–α corner in ΦCD508. We are currently testing the role of the X-loop through genetic engineering of *C. difficile* phages.

### 4.7. The role of the sheath in driving target envelope penetration

CISs store elastic energy in the metastable extended helical sheath. In these systems, the extended conformation is stabilized by interwoven protein meshes – which include various noncovalent subunit interactions such as the characteristic β-strand handshakes [Figs. 6 ▸(*d*) and 6 ▸(*e*)] – this potential energy is converted into kinetic energy upon contraction. Baseplate changes triggered by host recognition cause a domino-like collapse of the sheath, driving coordinated rotation, translation, and tube extension that mechanically drives the tube through the host-cell envelope.

Direct experimental measurements of force and velocity in CISs are few; most quantitative data are derived from modelling or indirect estimates. Estimates of the stored energy released in CISs include, for example, the extended T4 phage tail, ranging from ∼6000 to ∼15 000 kT (Maghsoodi *et al.*, 2017 ▸, 2019 ▸; Arisaka *et al.*, 1981 ▸); an R-type pyocin, ∼3500 kT (Fraser *et al.*, 2021 ▸); and in antifeeding prophage, ∼1200 kT (Desfosses *et al.*, 2019 ▸). We have not yet attempted to estimate the energy release for ΦCD508, but it is noteworthy that a *PISA* analysis (Krissinel & Henrick, 2005 ▸), based on the change in buried surface area of tail subunits when the tail contracts, gives a small increase in ‘free energy’ (2.3 kcal mol^−1^). This is a consequence of the significantly reduced contraction length, thus raising the question of what is the main energetic driver of envelope penetration by this phage (Wilson *et al.*, 2025 ▸). The need to understand this becomes even more compelling given the absence of lytic enzymes. Similarly, using the available coordinates for diffocin (Cai *et al.*, 2024 ▸) we calculate a decrease in buried surface area upon contraction, which would likely also result in a calculated ‘free energy’ increase.

For ΦCD508, a partial clue may lie in the tail tube protein, gp56, which lacks specific loops (α-loop and N-loop) found in other myovirus tail tube proteins (Wilson *et al.*, 2025 ▸) [Figs. 1 ▸(*c*) and 1 ▸(*e*)]. This omission results in more open packing between rings of the tail tube, which may thus allow the phage to bend in the contracted state. When we compare the tube protein of diffocin with that of ΦCD508 [Figs. 1 ▸(*a*), 1 ▸(*c*) and 1 ▸(*e*)], the ΦCD508 tube seems more ‘siphovirus-like’; therefore, does ΦCD508 adopt some characteristics of siphovirus envelope penetration? Siphoviruses generate no contractile force but may exploit the flexible nature of the central fibre to optimize the ability of the phage to explore a larger volume for penetration through the envelope, assisted by pressure from the tightly packed DNA in the viral head. Is this why cell-wall hydrolases are not required in ΦCD508? Moreover, we previously noted that the post-contraction ΦCD508 tail is unusually flexible, possibly as a result of this looser packing in the tail tube compared with other myophages; this could contribute to an entropic gain and by implication free energy decrease even though there is little change in buried surface area of the sheath subunits (Wilson *et al.*, 2025 ▸). Cai *et al.* also note the contribution of additional disorder in the sheath during contraction, presumably through increased conformational freedom in the post-contraction state (Cai *et al.*, 2024 ▸). This increased disorder and flexibility in the post-contraction state would presumably contribute to an entropic gain. A similar gain in ΦCD508 (reflected in the lower overall resolution of the contracted-state reconstruction) could thus further facilitate the apparently purely mechanical action required for tube ejection and penetration into the target cell.

Another remaining question is on the role of domain III in the sheath, since diffocin can penetrate the host envelope without it (Cai *et al.*, 2024 ▸) [Fig. 1 ▸(*d*)]. This domain appears very disordered in both extended and contracted forms (Fig. S2); it does not form direct contacts with neighbouring sheath subunits and thus will not contribute to any change in binding enthalpy upon contraction. We might speculate that it has some role in modulating the contraction and penetration mechanism; could the domain contribute to a viscous drag, providing overdamping to prevent spring-like oscillations on contraction and/or lowering the entry velocity of the tube? Perhaps this is important to avoid lethal damage to the host cell, which, by contrast, is an unimportant consideration in diffocin action.

### 4.8. Overall comparison of tape-measure predicted folds

We predict the tape measure to be largely α-helical with a similar predicted topology to that of other CISs (Wilson *et al.*, 2025 ▸). Some segments are predicted to fold into globular domains of short supercoiled helices, although they could not adopt such folds whilst constrained within the phage tail; similar segments are found with the diffocin tape measure (Cai *et al.*, 2024 ▸), although we have found no compelling match in fold of these domains between the two proteins (data not shown). These domains are of unknown function, but presumably their folding once released from the tail would contribute additional stability to the collapsed tail state. A notable point is that the diffocin tape measure is predicted to have a five-helix TM domain (Cai *et al.*, 2024 ▸) whilst that of ΦCD508 is predicted to have only three helices (Wilson *et al.*, 2025 ▸); this suggests that the diffocin could form a wide TM pore for lethal dissipation of the membrane potential whilst ΦCD508 forms a narrow sealed pore for DNA insertion that prevents, for example, proton leakage – a prerequisite for maintaining a viable host for prophage insertion or phage replication.

Through our image processing workflow, the full *in situ* structure of the TMP of ΦCD508 is not revealed as different segments along the length of the tail have been averaged to enhance the signal from the sheath and tube – the TMP does not have the same repetitive helical symmetry. However, we have revealed the structure of the C-terminal end in our analysis of the baseplate–needle complex as three splayed-out α-helices (Wilson *et al.*, 2025 ▸), confirming that the TMP is most likely trimeric [Fig. 3 ▸(*a*)]. There are few examples where the *in situ* TMP structure has been described in any detail but notable exceptions include the diffocin structure of Cai *et al.* and that of an archaeal virus, HFTV1, although in neither case was resolution sufficient to build a full atomic model along the whole length (Cai *et al.*, 2024 ▸; Zhang *et al.*, 2025 ▸). As for the diffocin, we see splayed-out C-terminal helices at the interface between the TMP and the spike/needle trimer in ΦCD508. The HFTV1 *in situ* TMP is hexameric, with three of the six C-terminal domains appearing to form a similar splayed-out structure.

## 5. Comparative receptor recognition and host specificity

### 5.1. The host S-layer

**Figure 7:**
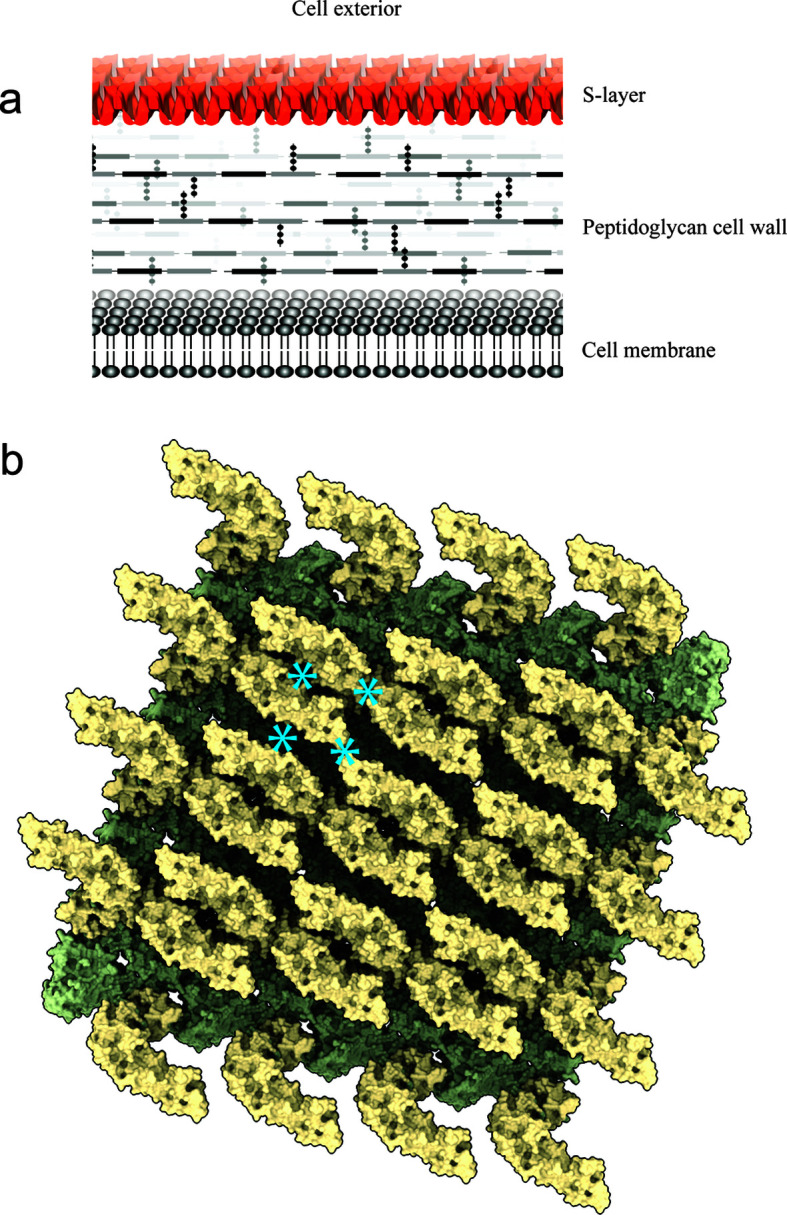
(*a*) Schematic diagram of a section through the *C. difficile* cell envelope. (*b*) Surface rendering of a model of a section of *C. difficile* S-layer viewed from outside the cell, with tightly packed SlpA subunits (PDB ID 7acy; Lanzoni-Mangutchi *et al.*, 2022 ▸). HMW SlpA, green; LMW SlpA, cream; axes of twofold symmetry, blue stars. It is notable that the openings in the S-layer are not wide enough for the passage of either a phage or diffocin tail tube.

The S-layer protein A (SlpA) is the primary structural component of the *C. difficile* S-layer, a paracrystalline protein array that coats the entire bacterial cell (Fagan & Fairweather, 2014 ▸; Isbilir *et al.*, 2025 ▸). It is critical for cell integrity, host adhesion and immune evasion. SlpA is synthesized as a single precursor polypeptide; during secretion, the precursor is cleaved by a cell-wall-associated cysteine protease, Cwp84. This cleavage yields two distinct subunits: a surface-exposed low molecular weight (LMW) subunit and a cell-wall-anchoring high molecular weight (HMW) subunit (Fig. 7 ▸). These two subunits remain non-covalently but tightly associated to form the basic building block of the S-layer, known as the H/L complex (Fagan *et al.*, 2009 ▸; Lanzoni-Mangutchi *et al.*, 2022 ▸). The two subunits have distinct structural roles and conservation profiles.

The HMW subunit acts as the anchor of the S-layer. It is positioned closer to the peptidoglycan cell wall, forming the base of the array, and contains three tandem CWB2 (cell-wall binding 2) motifs. These motifs bind specifically to the secondary cell-wall polymer (PSII), a polysaccharide composed of anionic teichoic acid-like polymers (Willing *et al.*, 2015 ▸). The sequence of the HMW subunit is highly conserved across different *C. difficile* strains, ensuring a common anchoring mechanism.

The LMW subunit acts as the interaction interface. It is oriented toward the external environment, on the outer surface of the cell envelope. It is composed of two domains: Domain 1 (D1), involved in interacting with the HMW subunit, and Domain 2 (D2), which extends outward and is the most surface-exposed region. The LMW subunit is highly variable between strains. This antigenic variability defines the specific S-layer cassette type (SLCT) of the strain. *C. difficile* strains are categorized into 14 distinct SLCTs, although there are likely to be many more types that have yet to be described.

The H/L complexes self-assemble into a 2D paracrystalline array covering the bacterium. The HMW subunits tile together to form triangular prisms that sit directly on the cell wall (Fig. 7 ▸). The LMW subunits project outward from these prisms, forming ridges that face the environment. Unlike many other bacterial S-layers, which function as coarse molecular sieves with large pores (3–10 nm), the *C. difficile* S-layer is exceptionally tight. The pores are very small (∼1 nm), which likely has consequences for phage penetration (see below).

### 5.2. Receptor-binding proteins compared

The selective action of both diffocins and bacteriophages stems from their targeted recognition of the highly variable S-layer (Whittle *et al.*, 2022 ▸; Royer *et al.*, 2023 ▸; Kirk, Gebhart *et al.*, 2017 ▸) through RBPs [Fig. 1 ▸(*a*)]. The polymorphic LMW component of SlpA is a determining factor for targeted binding (Kirk, Gebhart *et al.*, 2017 ▸) (Fig. 7 ▸). While some phages have a relatively broad host range, often concentrated within a few lineages, many others are highly restricted, infecting only one or a few strains. Thus, sequence variability, particularly in the exposed LMW portion, appears to dictate the narrow host range observed in many *C. difficile* phages (Fagan *et al.*, 2009 ▸; Royer *et al.*, 2023 ▸).

**Figure 8:**
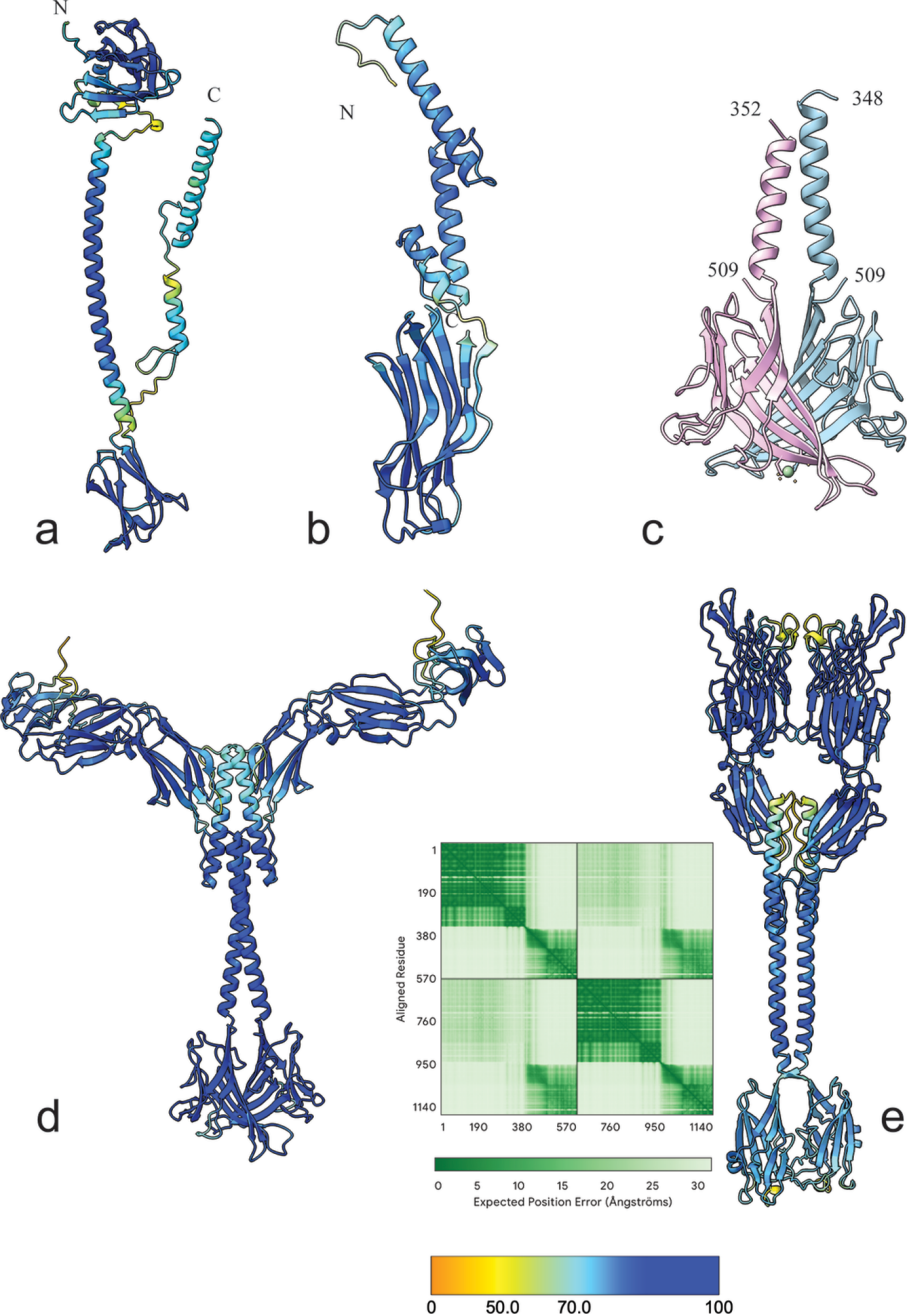
(*a*) *AlphaFold3* model of ΦCD508 gp67, a putative baseplate attachment protein forming part of the tail fibre. (*b*) *AlphaFold3* model of ΦCD508 gp68, a putative RBP of the tail fibre assembly. (*c*) X-ray crystallographic structure of the putative receptor-binding domain of PtsM from Φ027. Individual chains are coloured along with a coordinated metal ion (green). (*d*) *AlphaFold3* model of a putative full-length PtsM dimer from Φ027. The C-terminal putative receptor-binding domain adopts the same fold as the experimental structure in (*c*). (*e*) *AlphaFold3* model of dimeric Pts_HN10_M; the putative RBP domain is at the bottom. The coloured scale bar indicates pLDDT (predicted local distance difference test) values.

RBPs are mostly found on tail fibres connected to the base plate. Resolving the full-length structure of RBPs remains challenging due to their inherent flexibility and elongated architecture. However, with deep-learning-based structure prediction tools, computational models have been generated for complete tail fibres, including RBPs. RBPs are modular, and domains can be exchanged between different phages via horizontal gene transfer (Pas *et al.*, 2023 ▸; Smug *et al.*, 2023 ▸; Haggård-Ljungquist *et al.*, 1992 ▸). Typically, an RBP has a conserved N-terminal part that binds to the phage baseplate, while the C-terminal tip is more variable; it either binds to the host receptor or connects to other RBPs, forming a complex (Yap & Rossmann, 2014 ▸; Bartual *et al.*, 2010 ▸; Klein-Sousa *et al.*, 2025 ▸). Annotation of *C. difficile* phage structural cassettes generally indicates at least two tail fibre proteins; for example, for ΦCD508 using *PHAROKKA* and *PHANOTATE* (Bouras *et al.*, 2023 ▸; McNair *et al.*, 2019 ▸), we have identified two putative tail fibre proteins, gp67 and gp68 [Fig. 8 ▸ (*a*) and 8 ▸(*b*)]. The protein gp68 is likely to contain the receptor-binding domain; we hypothesize gp67 to be a linker protein [baseplate attachment protein (Gebhart *et al.*, 2015 ▸)] attached at one end to the baseplate and to gp68 at the other. Further evidence for this role for gp68 comes from analyses of putative tail fibres from other *C. difficile* phages, which indicate a protein of similar conserved fold even when the cognate RBP is different (data not shown). The structure determined by Cai *et al.* (2024 ▸) is indeed of an engineered diffocin, where the C-terminal end of the baseplate attachment protein was engineered to be compatible with a phage RBP (Gebhart *et al.*, 2015 ▸).

In contrast to the conserved gp67 type structure, there appears to have been a co-evolution of the unique fold of the SlpA LMW domain II and a variety of specialized RBP structures. While phage RBPs are predicted to be structurally diverse (Fig. 8 ▸), experimental data are extremely limited. One crystal structure for a non-contractile *C. difficile* siphophage RBP has been reported (Dowah *et al.*, 2021 ▸) but until now no structure from a contractile *C. difficile* phage has been available. Here we report on the crystallization and structure determination of the putative RBP PtsM from Φ027 (Gebhart *et al.*, 2015 ▸) to 1.4 Å resolution by X-ray diffraction [Fig. 8 ▸(*c*)]. The structure contains the C-terminal residues that make up the RBP domain; they form an unusual C2 dimeric assembly; to our knowledge, this is unlike any other described RBPs, which are typically trimeric. Each monomer is formed of mostly β-strands, assembled to form two sheets. The larger sheets form a dimeric interface and assemble into a bell-like structure, with loops protruding from the open end. An unidentified metal ion is octahedrally co-ordinated in the centre of the structure, emphasizing the bell-like construction. We hypothesize that the unusual C2 dimeric assembly is optimized to interact with the twofold symmetry of the assembled S-layer (Lanzoni-Mangutchi *et al.*, 2022 ▸) (Fig. 7 ▸). An *AlphaFold3* (Abramson *et al.*, 2024 ▸) prediction for a full-length sequence appears compatible with this dimeric arrangement [Fig. 8 ▸(*d*)].

Pts_HN10_M, identified as a putative RBP from the prophage of *C. difficile* strain HN10, was interpreted to be a stable homotrimer in its native form, based on gel filtration and cross-linking experiments. Structural mapping of truncated variants confirmed that the C-terminal domain is solely responsible for binding to the *C. difficile* cell surface and specifically interacting with the LMW subunit of SlpA (Phetruen *et al.*, 2022 ▸). *AlphaFold3* modelling indicates that a dimeric arrangement is more probable, with particular confidence in the C-terminal RBP domain [Fig. 8 ▸(*e*)]. The RBP domain fold does not match that of PtsM from Φ027 described above.

**Figure 9:**
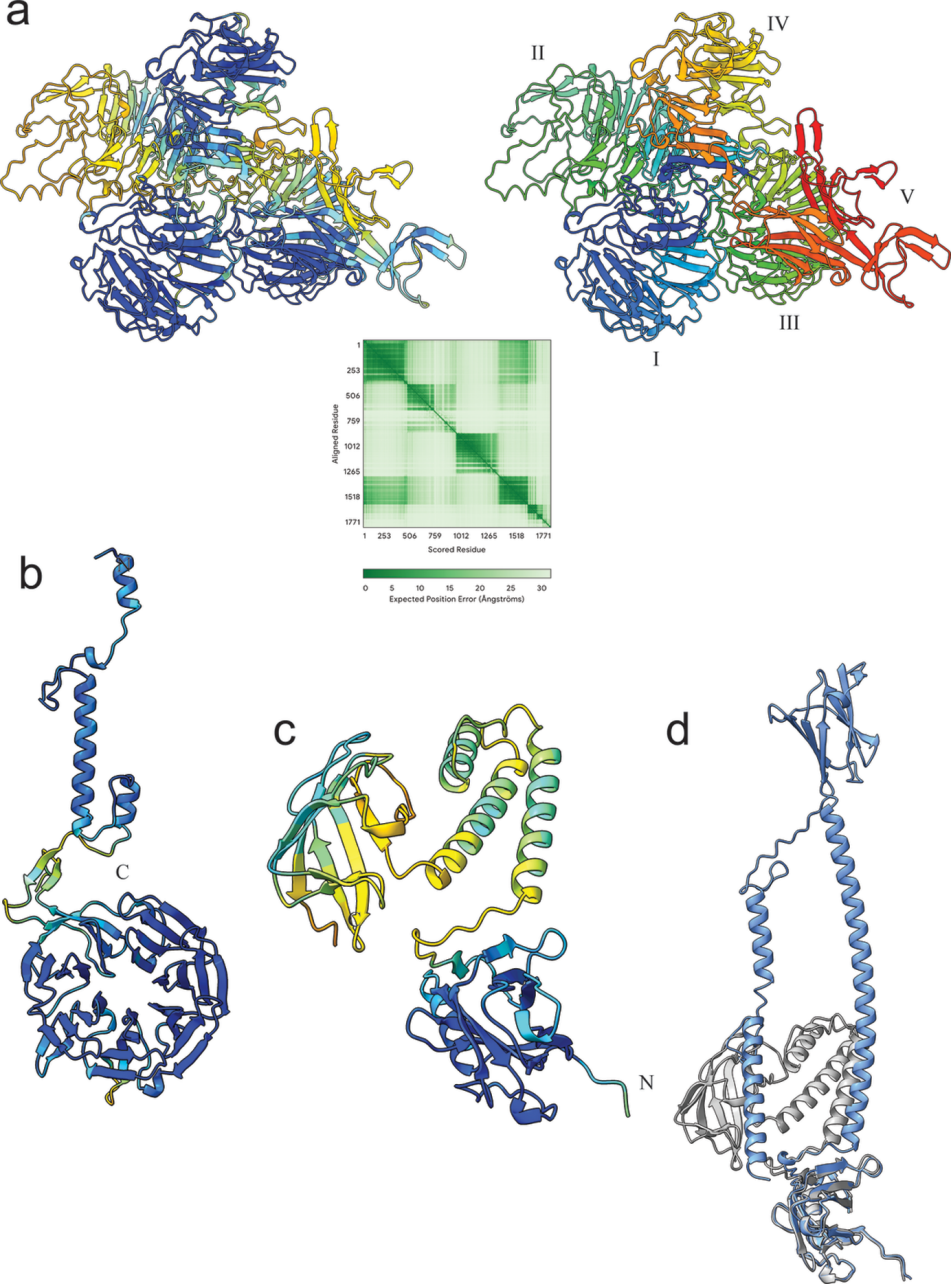
(*a*) *AlphaFold3* model of RbtM, a putative RBP, from diffocin-4 (Gebhart *et al.*, 2015 ▸). The predicted aligned error, dark green, for residues in domain I (residues 1 to 385) compared with domain IV (residues 1292 to 1558) is relatively low as they are predicted to bind together through a shared β-sheet as seen in the rainbow-coloured model (N-terminus, blue; C-terminus, red). (*b*) *AlphaFold3* model of gp33 from *C. difficile* phage ΦMMP03. (*c*) *AlphaFold3* model of RtbL, a putative baseplate attachment protein from diffocin-4 (Gebhart *et al.*, 2015 ▸). Note that the apparent helical bundle is predicted with low confidence and could be stretched into one long helix as predicted for gp67 of ΦCD508 [see (*d*)]. (*d*) Alignment of predicted structures of ΦCD508 gp67 and diffocin-4 RtbL; close alignment is seen for the N-terminal domains.

In diffocins, the RBP is often identified as a very large protein (∼200 kDa) that serves as the primary determinant of the killing spectrum (Gebhart *et al.*, 2015 ▸, 2012 ▸; Kirk, Gebhart *et al.*, 2017 ▸). No structures are currently available, however an *AlphaFold3* prediction of the 3D structure of the RtbM putative RBP sequence (UniProt A0A1X9K4E9) indicates a multidomain structure including three WD40 domains [Fig. 9 ▸(*a*)].

A near-identical fold to that of the predicted ΦCD508 RBP, gp68, is predicted for some other contractile *C. difficile* phages, including ΦMMP03 (NCBI Reference Sequence: NC_028959.1) and CDKM15 (Rashid *et al.*, 2016 ▸). The multimeric state of these predicted RBPs is unclear, with ipTM (interface predicted template modeling) scores relatively low for multimeric states up to four (data not shown). Annotation of the structural cassette within the ΦMMP03 and CDKM15 genomes indicates a second potential tail fibre protein with a predicted WD40 fold [Fig. 9 ▸(*b*)]. This is reminiscent of domains found in the diffocin RBP [Fig. 9 ▸(*a*)]. WD40 domain proteins are relatively rare in bacteria but typically form platforms for interaction with other proteins (Smith *et al.*, 1999 ▸), suggesting a likely role in S-layer protein recognition. It is unclear what different roles the two types of RBP may play. One may play a role in transient initial binding followed by final irreversible binding mediated through a second one, as proposed for T4 phage (Hu *et al.*, 2015 ▸). Another possibility is that one recognizes the main SlpA protein (dimeric RBPs) and the other recognizes a second receptor, perhaps one of the other minor cell-wall proteins (Fagan & Fairweather, 2014 ▸). Further work needs to be done to test these ideas.

Thus, a partial search of selected *C. difficile* phage genomes indicates a variety of unrelated folds for the RBPs, which likely reflect the different evolutionary strategies to adapt to different SlpA variants. In contrast to this, the proteins likely to link the RBPs to the baseplate are less variable, and all phages discussed above encode a protein homologous to gp67 of ΦCD508 with a predicted fold shown in Fig. 8 ▸(*a*). *AlphaFold3* analysis of the RtbL protein from diffocin-4 (Gebhart *et al.*, 2015 ▸) shows a very similar N-terminal fold followed by a similar long α-helical domain. However, the C-terminal region, including the β-sheet domain, deviates in structure from the ΦCD508 gp67-type fold [Figs. 9 ▸(*c*) and 9 ▸(*d*)]. Thus, it seems likely that the structurally conserved N-terminal domain is responsible for attachment to the baseplate, whilst the more variable C-terminal domain is adapted to interact with different types of RBP; this is consistent with findings by Gebhart *et al.*, who constructed a functional fusion of the N-terminal portion of the diffocin-4 baseplate linker RtbL with the C-terminal portion of the Φ027 baseplate linker PtsL and the Φ027 RBP, PtsM (Gebhart *et al.*, 2015 ▸). Presumably, this modular arrangement allows phages to rapidly adapt to new hosts by optimizing the C-terminal receptor-binding element without compromising the stability of the N-terminal tail assembly components.

No experimental atomic structure is available for any *C. difficile* phage or diffocin baseplate attachment protein, even though tail fibres were present in both the ΦCD508 and diffocin particles subjected to cryoEM analyses (Wilson *et al.*, 2025 ▸; Cai *et al.*, 2024 ▸). This indicates that the tail fibres are connected to the baseplate rather flexibly and thus poorly resolved in the reconstructions. In our single-particle analysis of ΦCD508, a low-pass filtered reconstruction indicates the approximate envelope of at least part of gp67 [Fig. 3 ▸(*b*)]. We also observed tail fibres/RBPs attached to S-layer fragments in tomograms determined by electron cryotomography (cryoET) (Wilson *et al.*, 2025 ▸). The visible parts of these appear as straight rods running perpendicular to the S-layer surface, with a length of ∼27 nm; the length of gp68 [Fig. 8 ▸(*b*)] will be of the order of 10 nm at most, so the additional length must be conferred by the attached gp67 baseplate attachment protein [Fig. 8 ▸(*a*)]; the *AlphaFold3* prediction is for gp67 to have two long α-helical segments that could run to a full length of up to the order of 16 nm. Thus, the apparent tail fibre length measured from tomograms of receptor-bound phages is consistent with the gp67/gp68 complex being made up of long stretches of α-helices, probably arranged as multimeric bundles.

## 6. Conclusions: a framework for engineering precision antimicrobials

The resolution revolution in cryoEM has fundamentally transformed virus research (Schoehn *et al.*, 2023 ▸); as in other areas of structural biology, we have moved from ‘blobology’ to atomic-level structural biology. For decades, structural biol­ogists have exploited symmetry averaging to solve phage structures; for example, phage capsids are often icosahedral, so averaging thousands of images can lead to a substantially enhanced signal. However, this approach smears out unique components of lower symmetry, such as the portal vertex (the machinery that pumps DNA in and out of the capsid). With more recently developed approaches such as focused classification and symmetry relaxation, we can now reconstruct these unique parts without averaging them away.

From low-resolution negative stain EM to X-ray crystallography, which required crystallizing purified proteins and thus taking the phage apart, to now with cryoEM, we can analyse the whole virus interacting with a bacterial cell in molecular detail. Some of the most dramatic functional insights have come from solving the structures of multi-component complexes such as the baseplate and tail in different states (Cai *et al.*, 2024 ▸; Wilson *et al.*, 2025 ▸; Li *et al.*, 2023 ▸; Taylor *et al.*, 2016 ▸; Xu *et al.*, 2026 ▸). In the last few years, cryoEM has even captured phages in the act of infection on the bacterial surface (Zhou & Lok, 2024 ▸; Wilson *et al.*, 2025 ▸; Hu *et al.*, 2015 ▸). However, there are still challenges remaining; although tail fibres have been largely invisible to X-ray crystallography because of their flexible nature, they also remain difficult for cryoEM (Mourosi *et al.*, 2022 ▸). Nevertheless, advances in tomography should help and we have already made a start on this (Wilson *et al.*, 2025 ▸).

In the specific case study described here, the comparative analysis of the ΦCD508 and diffocin baseplates provides fundamental insights into the structural constraints and adaptations required for successful breaching of the Gram-positive bacterial cell envelope. The key structural difference lies in the penetration mechanics: diffocin adopts a combined chemical and mechanical approach via the hub-hydrolase (CD1368), while ΦCD508 appears to operate through pure mechanical thrust, possibly facilitated through the flexible tail tube assembly. Both systems converge, however, on utilizing metal coordination chemistry to maximize the rigidity of the central puncturing spike. The mechanism by which the tightly packed S-layer is breached remains a mystery, although we have speculated on a number of possible solutions, such as penetration at crystal defects with the S-layer array; these ideas need to be tested in the future (Wilson *et al.*, 2025 ▸).

The resolution revolution in cryoEM will move the field beyond simply finding the right RBP to understanding the entire sequence of mechanical events that follow attachment. By visualizing the infection machinery as a dynamic series of ‘molecular switches’, we can look forward to engineering phages that are not just better at binding, but better at injecting and surviving hostile environments. For example, we may be able to manipulate the trigger sensitivity of the baseplate, which undergoes a large conformational change (Xu *et al.*, 2026 ▸), releasing the sheath strands (Yap *et al.*, 2016 ▸). The baseplate acts as a ‘safety catch’, which could potentially be engineered to lower the activation energy barrier so that particles fire more easily. Conversely, for therapeutic storage, we might wish to stabilize the baseplate trigger to prevent spontaneous firing – as we have observed in ΦCD508 preparations [Fig. 6 ▸(*a*)]. Opportunities for the construction of chimeric phages will emerge, for example, by swapping entire capsid and tail assemblies between phages, combining the best mechanical properties of components – something impossible without atomic-level knowledge.

It is unclear how critical the enzymatic hub found in diffocin is for cell-wall penetration, so we are currently investigating the structures of *C. difficile* phages that are predicted to assemble cell-wall hydrolases into the virion. How and when hydrolases are presented to the cell wall during the penetration process deserves further investigation; future structural studies should focus on utilizing cryoET to observe intermediate firing states. Tail-contraction mechanisms need further elucidation, with the coordination between tail contraction and genome release still poorly understood [Fig. 6 ▸(*a*)].

## Supplementary Material

Supporting figures. DOI: 10.1107/S2052252526005105/rq5019sup1.pdf

## Figures and Tables

**Table 1:** Helical parameters for ΦCD508 tail conformations

Form	Twist	Rise	Symmetry
Extended (consensus)	+19.2°	38.9 Å	C6
Extended (selected from 3DVA, approximate helical parameters)	−40.8°	38.9 Å	Quasi-C3
Contracted urea	+26.1°	31.8 Å	C6
Contracted without urea	+25.5°	32.4 Å	C6

## Data Availability

X-ray and cryoEM model coordinates will be deposited in RCSB PDB. CryoEM maps will be deposited in the EMDB.
