# Supplementary material for: How cryoEM has advanced our understanding of bacteriophages and bacteriocins targeting Clostridioides difficile
Source: IUCrJ. 2026 Jun 23;13(Pt 4):324–42. doi: 10.1107/S2052252526005105 (PMC13324602; doi:10.1107/S2052252526005105)
Supplement: Supplementary file 1 [file m-13-00324-sup1.pdf]

IUCrJ

Volume 13 (2026)

Supporting information for article:

How cryoEM has advanced our understanding of bacteriophages and bacteriocins targeting *Clostridioides difficile*

Per A. Bullough, Jason S. Wilson, Hannah L. Berry and Robert P. Fagan

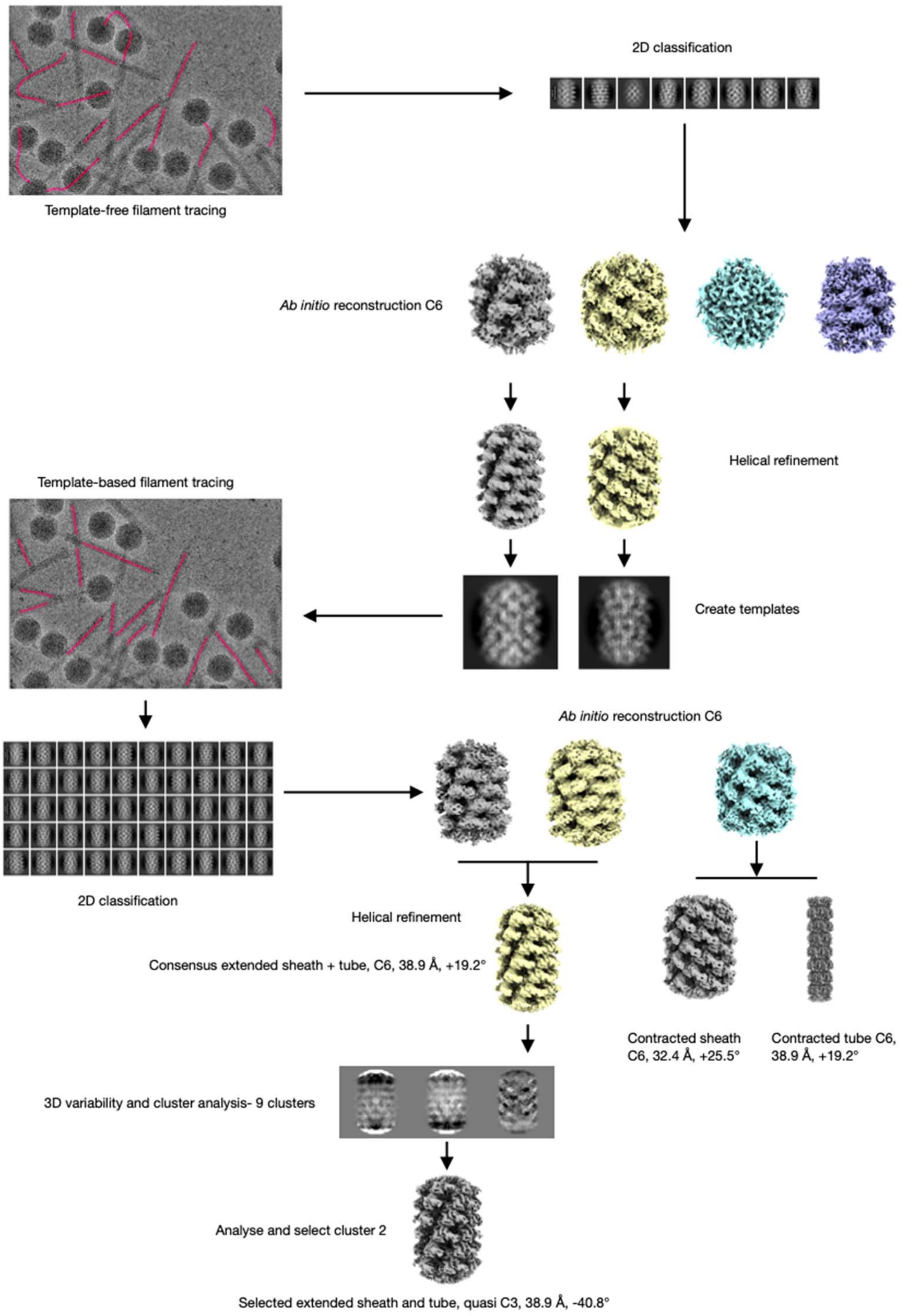

Figure S1 Workflow for image processing of ΦCD508 tails from samples containing extended and spontaneously contracted sheaths.

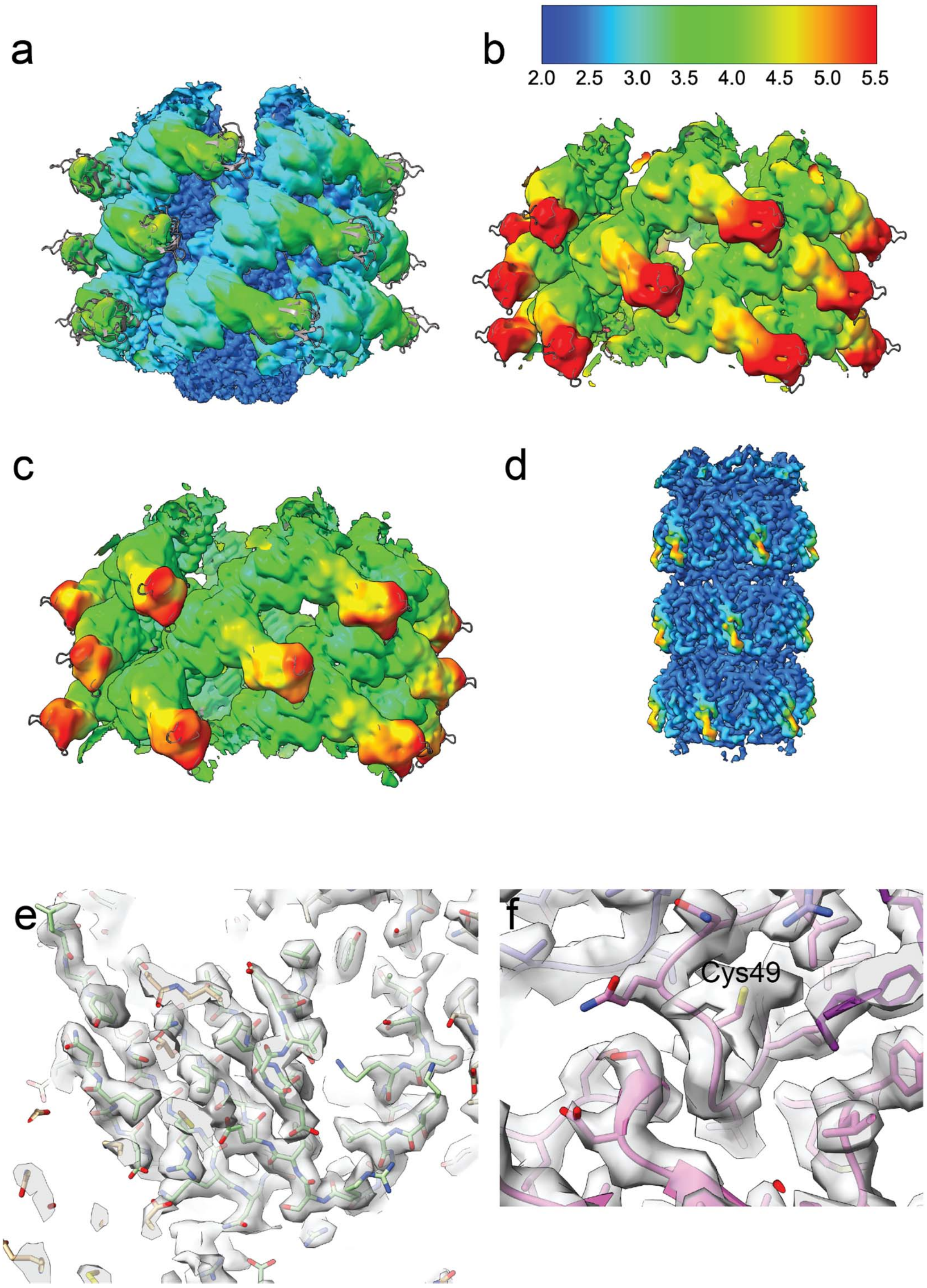
a
b
2.0 2.5 3.0 3.5 4.0 4.5 5.0 5.5
c
d
e
f
Cys49

Figure S2 (a-d) Unsharpened density maps coloured according to resolution with atomic models fitted. Surface contour thresholds have been chosen arbitrarily. The color key corresponds to resolution in Å. (a) Reprocessed extended tail sheath and tube. (b) Reprocessed urea-induced contracted tail sheath. (c) Naturally contracted tail sheath. (d) Naturally contracted tail tube. (e) Representative sharpened density and model fit from the tube in (a). (f) Density around tube protein cys49, in the extended tail, showing additional density in the side chain. This may indicate an oxidised state. It is notable that this residue is located where three separate subunits meet. A similar density is seen in the tube of the contracted tail.
